# A comparison of marker-based estimators of inbreeding and inbreeding depression

**DOI:** 10.1186/s12711-022-00772-0

**Published:** 2022-12-27

**Authors:** Armando Caballero, Almudena Fernández, Beatriz Villanueva, Miguel A. Toro

**Affiliations:** 1 Centro de Investigación Mariña, Universidade de Vigo, Facultade de Bioloxía, 36310 Vigo, Spain; 2 Departamento de Mejora Genética Animal, INIA-CSIC, Ctra. de La Coruña, Km 7.5, 28040 Madrid, Spain; 3 Departamento de Producción Agraria, ETSI Agronómica, Alimentaria y de Biosistemas, Universidad Politécnica de Madrid, 28040 Madrid, Spain

## Abstract

**Background:**

The availability of genome-wide marker data allows estimation of inbreeding coefficients (*F*, the probability of identity-by-descent, IBD) and, in turn, estimation of the rate of inbreeding depression (ΔID). We investigated, by computer simulations, the accuracy of the most popular estimators of inbreeding based on molecular markers when computing *F* and ΔID in populations under random mating, equalization of parental contributions, and artificially selected populations. We assessed estimators described by Li and Horvitz (*F*_*LH*1_ and *F*_*LH*2_), VanRaden (*F*_*VR*1_ and *F*_*VR*2_), Yang and colleagues (*F*_*YA*1_ and *F*_*YA*2_), marker homozygosity (*F*_*HOM*_), runs of homozygosity (*F*_*ROH*_) and estimates based on pedigree (*F*_*PED*_) in comparison with estimates obtained from IBD measures (*F*_*IBD*_).

**Results:**

If the allele frequencies of a base population taken as a reference for the computation of inbreeding are known, all estimators based on marker allele frequencies are highly correlated with *F*_*IBD*_ and provide accurate estimates of the mean ΔID. If base population allele frequencies are unknown and current frequencies are used in the estimations, the largest correlation with *F*_*IBD*_ is generally obtained by *F*_*LH*1_ and the best estimator of ΔID is *F*_*YA*2_. The estimators *F*_*VR*2_ and *F*_*LH*2_ have the poorest performance in most scenarios. The assumption that base population allele frequencies are equal to 0.5 results in very biased estimates of the average inbreeding coefficient but they are highly correlated with *F*_*IBD*_ and give relatively good estimates of ΔID. Estimates obtained directly from marker homozygosity (*F*_*HOM*_) substantially overestimated ΔID. Estimates based on runs of homozygosity (*F*_*ROH*_) provide accurate estimates of inbreeding and ΔID. Finally, estimates based on pedigree (*F*_*PED*_) show a lower correlation with *F*_*IBD*_ than molecular estimators but provide rather accurate estimates of ΔID. An analysis of data from a pig population supports the main findings of the simulations.

**Conclusions:**

When base population allele frequencies are known, all marker-allele frequency-based estimators of inbreeding coefficients generally show a high correlation with *F*_*IBD*_ and provide good estimates of ΔID. When base population allele frequencies are unknown, *F*_*LH*1_ is the marker frequency-based estimator that is most correlated with *F*_*IBD*_, and *F*_*YA*2_ provides the most accurate estimates of ΔID. Estimates from *F*_*ROH*_ are also very precise in most scenarios. The estimators *F*_*VR*2_ and *F*_*LH*2_ have the poorest performances.

**Supplementary Information:**

The online version contains supplementary material available at 10.1186/s12711-022-00772-0.

## Background

The characterization and management of inbreeding are important topics in conservation [1] and in population and quantitative genetics [2–4]. Proper estimation of individual inbreeding coefficients is necessary to estimate inbreeding depression, i.e., the change in the mean of a quantitative trait, which mainly results from the expression of recessive alleles with their effect being hidden in heterozygotes and exposed in homozygotes by inbreeding (see [2], Chapter 10). The inbreeding coefficient of an individual is defined as the correlation between homologous alleles [5] or the probability of identity-by-descent (IBD) of homologous alleles [6], and has been traditionally obtained from pedigree data [7]. However, inbreeding can also be obtained from genomic information (see e.g., [8]) and the development of high-density single nucleotide polymorphism (SNP) panels for an increasing number of species gives the opportunity to develop and implement methods that can accurately estimate the true realized inbreeding [9]. Genomic information also enables the levels of genetic variation across the genome to be evaluated [10–12] or the identification of genomic regions that are responsible for inbreeding depression [13, 14].

There are many metrics to estimate relatedness and inbreeding from marker data that are applied on a SNP-by-SNP basis and need the knowledge of marker allele frequencies [15–24]. Another useful marker-based measure to estimate inbreeding is the proportion of the autosomal genome that is composed of runs of homozygosity (ROH) [25], as well as other alternative analogous metrics [26]. ROH measures of inbreeding provide estimates of IBD and have been shown to be useful estimators of the inbreeding coefficient and as a basis for estimating inbreeding depression [9, 27, 28] but their performance depends on the criteria used to define a ROH [29, 30]. Marker frequency-based measures of inbreeding are expected to provide unbiased estimates of the average IBD measures of relatedness and inbreeding relative to an initial generation or base population that is assumed to consist of non-inbred and unrelated individuals, when the allele frequencies in that initial generation are known [19, 22, 31, 32]. However, allele frequencies in the base population are usually not available and inferring them is difficult [33], thus frequencies in the current generation are often used instead. In that case, marker frequency-based measures of inbreeding provide deviations from Hardy–Weinberg proportions or correlations of allele frequencies of homologous genes relative to the current generation of the population rather than estimates of IBD [22], and the estimates are confounded by the average coancestry of individuals with other individuals in the sample taken for analysis [34].

The different measures of molecular inbreeding have been evaluated in multiple studies, both based on empirical data [13, 14, 24, 34–44], and based on simulated data or theoretical analyses [9, 32, 34, 45–47]. Some empirical studies have used inbreeding estimators based on known [12] or estimated [35] allele frequencies from a predefined base population. In other studies, a frequency of 0.5 is assumed for all SNP alleles, which results in inbreeding estimates that have intermediate to high correlations with pedigree-based estimates or IBD [35, 37, 48]. Nevertheless, in most studies, allele frequencies in the current population are used to obtain different marker-based inbreeding estimates and these are compared with each other and with pedigree-based inbreeding estimates, with rather contrasting results. For example, the correlation of coefficients of inbreeding obtained from pedigrees with those obtained from markers is often intermediate ranging from 0.4 to 0.8 [13, 14, 39, 41, 42, 49], but can sometimes be low or even negative [39, 41, 44, 50, 51].

Previous simulation studies have investigated the accuracy of different molecular measures of inbreeding to estimate IBD. One of the most comprehensive studies is that from Wang [23], who showed that when the number of markers is large enough (around 10,000), marker-based estimates of inbreeding are more correlated with IBD than pedigree-based estimates. A later simulation study by Forutan et al. [48], who extended the analyses to artificially-selected populations, showed that marker-based inbreeding coefficients were close to the IBD values when the frequencies of the base population are known, as expected. However, when constant allele frequencies of 0.5 were used, marker-based coefficients overestimated IBD, although the correlation of marker-based estimates with IBD values was close to 1.

The accuracy of using molecular measures of inbreeding based on current allele frequencies to estimate the rate of inbreeding depression (ΔID) has also been addressed by different studies [9, 23, 32, 46, 47]. These studies have shown that marker-based measures of inbreeding and relatedness are more precise and more powerful to detect inbreeding depression than pedigree-based measures. In addition, simulation results have indicated that some estimators of molecular inbreeding, such as those obtained from the correlation of uniting gametes [20] and those from ROH, generally provide good estimates of ΔID but can give biased estimates in some cases. However, comprehensive studies are still needed to elucidate the performance of the use of different marker-based estimators to estimate ΔID under some scenarios with non-random contributions from parents to progeny. For example, in conservation programs it is common to equalize contributions from parents to offspring in order to maintain the highest genetic diversity [1]. In addition, many populations are subjected to artificial selection, where contributions from parents to progeny can be far from random.

The objective of this study was to evaluate how different estimators of molecular inbreeding perform as measures of IBD and how precise they are when estimating ΔID, with or without knowledge of allele frequencies in the base population of reference. We focus on the most popular marker-based estimators that make use of SNP allele frequencies or ROH [42–44, 51–58] and that are readily obtained by commonly used software such as PLINK [59, 60] and GCTA [36]. We carried out computer simulations that assumed a model of selection against deleterious mutations, with random or equal contributions from parents to progeny, or artificial selection for a neutral quantitative trait. We also analysed a pedigreed population of Iberian pigs where equalization of family sizes has been intended over 23 generations, and compared values of inbreeding and inbreeding depression obtained for the different measures of inbreeding based on a simulation of this population with their observed empirical values.

## Methods

### Simulation of populations under different breeding program designs

#### Large initial population

We used the software SLiM 3 [61] to simulate the genome of a large population of a sexually reproducing diploid species with random mating and discrete generations. As a model, we assumed the genomic characteristics of the pig genome [62], as some of the simulation results are compared with those obtained from empirical data of a strain of Iberian pigs (the Guadyerbas strain). A population with a constant size of *N* = 500 individuals was simulated for 5000 generations, which is the approximate effective population size estimated for the ancestral population of the Guadyerbas strain [63]. We assumed a total genome size of 2250 Mb, with 18 chromosomes of 125 Mb each. A summary of the main parameters used in the simulations is in Table 1. A recombination rate of 8 × 10^–9^ was assumed, with crossover frequencies following a Poisson distribution with a uniform probability across the genome and without interference. This implies a genome length of about 1 Morgan per chromosome and a recombination rate of 0.8 cM/Mb, which is close to that found in the pig genome [62]. The mutation rate per nucleotide was assumed to be 4 × 10^–9^ in order to obtain a number of SNPs close to that available for the pig population, but a model with a higher mutation rate (9 × 10^–9^) was also considered to investigate a higher density of SNPs.

**Table 1:** Simulation parameters for the default model, a model with a higher density of SNPs, and an alternative model of deleterious mutations with a lower mean effect than for the default model

	Default model	Higher density	Alternative model
Genome length (Mb)	2250	–	–
Number of chromosomes	18	–	–
Recombination rate	8 × 10^–9^	–	–
Mutation rate	4 × 10^–9^	9 × 10^–9^	–
Percentage of deleterious mutations	5	2.22	10
Average effect of mutations ($\overline{s }$)	0.1	–	0.025
Average dominance coefficient ($\overline{h }$)	0.2	–	–
Number of SNPs (/1000)			
*N* = 500	125	286	N/A
*N* = 20			
* t* = 0	65	147	N/A
*t* = 10	35	85	N/A
*t* = 20	25	N/A	N/A
*N* = 100			
*t* = 0	91	N/A	N/A
*t* = 50	38	N/A	N/A
*t* = 100	28	N/A	N/A

The hyphens indicate values equal to those of the default model, while N/A indicates scenarios not run

*N* number of breeding individuals, *t* generation number

In order to evaluate ΔID for fitness, it was assumed that 5% of the mutations were deleterious. The fitness of the wild type genotype, the heterozygote, and the mutant homozygote were 1, 1 − $sh$, and 1 − $s$, respectively, where the homozygous selection coefficient $s$ was obtained from an exponential distribution with mean $\overline{s }$ = 0.1, and the dominance coefficient $h$ was obtained from a uniform distribution between 0 and ${e}^{(-ks)}$, where $k$ was set to obtain an average value of $\overline{h }$ = 0.2 [4, 64]. Individual genotypic fitness values were obtained multiplicatively across loci. We also assumed an alternative model with a lower mean effect of deleterious mutations ($\overline{s }$ = 0.025) and a higher mutation rate (Table 1). The distribution of mutation effects and dominance coefficients assumed in the two models are shown in Additional file 1: Fig. S1.

#### Small populations with random contributions, equalization of parents´ contributions, or artificial selection

An in-house C program was used to simulate small populations of size $N$ = 20 or 100 breeding individuals over 10 (for $N$ = 20) or 50 (for $N$ = 100) discrete generations, such that the average inbreeding coefficient ($F$) in the last generation was about the same for both population sizes. Populations of size $N$ = 20 and $N$ = 100 were also simulated for 20 and 100 generations, respectively, in order to approximately double the average inbreeding coefficient in the last generation. To start these populations, individuals were randomly sampled from the large initial population of 500 individuals that was simulated as described previously. The first generation ($t$ = 0) was considered to be the base population that all inbreeding coefficients refer to. In one set of simulations, generation $t$ = 10 was considered the base population. The genome length, the number of chromosomes, the recombination rate between nucleotides, and the fitness effects were as described previously. It was assumed that the same SNP panel was used to compute measures of inbreeding in the initial and final generations. Thus, only SNPs that segregated at generation *t* = 0 were considered in the analyses and the mutation rate was set to zero for the subsequent generations. In order to estimate IBD probabilities, about 4500 multiallelic loci uniformly distributed across the genome were also simulated, with unique alleles assigned to each individual in generation $t$ = 0.

For the random contributions (RC) and equalization of parents´ contributions (EC) scenarios, a number $N$ of individuals were randomly taken from the large initial population to create each replicated line. For the RC scheme, parents were randomly mated to generate $N$ progeny, allowing polygamy but avoiding self-fertilization. For the EC scheme, the same procedure was followed, except that, in each generation, the $N$ individuals were randomly paired in couples and two offspring were obtained from each couple. For the artificial selection scheme (SEL), $T$ individuals (100 or 500) were taken at random from the large initial population to generate the base population of each replicate. From these individuals, $N$ (20 or 100, respectively) were artificially selected based on their own phenotype for a quantitative trait (described below), implying a selected proportion of 20%. Two thousand replicates were carried out for each simulated scenario.

The genetic basis of the quantitative trait was established as described in the following. From the SNPs of the large initial population, a random 5% were chosen as quantitative trait loci (QTL) and an effect on the quantitative trait was assigned to one of the two alleles. Effects were taken from a normal distribution with mean zero and standard deviation 0.1, assuming additive gene action. Additivity was also assumed between loci, and thus genotypic values were obtained as the sum of the effects of all QTL. The phenotypic value was obtained by adding an environmental deviation to the genotypic value, which was taken from a normal distribution with mean 0 and environmental variance 2, a value chosen such that it generated an initial heritability for the trait of around 0.5. The quantitative trait was assumed to be not related to fitness.

Pedigrees were recorded over generations to obtain the pedigree inbreeding coefficient of each individual in the last generation ($t$ = 10, 20, 50 or 100) to obtain an estimate of ${F}_{PED}$. The identity-by-descent inbreeding coefficient (${F}_{IBD}$) in the last generation was computed using the multiallelic loci. The genotypes of the SNPs (about 25,000–40,000 in the default model and more than twice as many in the higher density model; Table 1) were used to obtain the molecular inbreeding coefficients for each individual in the last generation. Loci that affected the selected quantitative trait or fitness were excluded when computing any measure of inbreeding. Marker allele-frequency based measures of $F$ (described below) were obtained using the known allele frequencies of the initial generation ($t$ = 0, considered the base population of reference), those of the last (current) generation ($t$ = 10 or 20 for $N$ = 20 and $t$ = 50 or 100 for $N$ = 100), or set to a constant value of 0.5. Runs of homozygosity were also obtained for individuals in the last generation. The mean and variance of the $F$ values of individuals in the last generation were obtained for each estimator of inbreeding. Pairwise Pearson’s correlations were also obtained between all inbreeding measures. Average estimates of inbreeding from the different molecular estimators were compared with the inbreeding coefficient obtained based on IBD values (${F}_{IBD}$) in the last generation.

Estimates of the rate of inbreeding depression (ΔID) were obtained for fitness as the regression coefficient of the logarithm of individual fitnesses in the last generation on the corresponding estimated inbreeding coefficients (see [2], p. 262). Additional file 2: Table S1 and Additional file 3: Fig. S2 illustrate the removal of deleterious mutations by genetic purging and drift, the reduction in the expected inbreeding depression in consecutive generations, and the change in additive and dominance variance for fitness for one of the simulated scenarios.

Power to detect inbreeding depression was obtained following Wang [23], by counting the percentage of replicates for which the value of ΔID was significantly different from zero with a probability *P* < 0.05. A bootstrap method based on 1000 resampling sets with replacement of the 2000 pairs of simulated estimates was used to compare the estimates of ΔID obtained from ${F}_{IBD}$ with those from the different molecular marker estimators. To confirm the bootstrap results, a paired sample *t*-test was also carried out using R Commander [65].

### Simulation of the Iberian pig population

The Guadyerbas population consists of a pedigreed population with 1206 individuals born across 23 cohorts, from which 219 (those born in cohorts 17–23) were genotyped with the Illumina Porcine SNP60 Bead Chip, which contains 35,519 SNPs that segregate in the Iberian breed [66]. Phenotypes for litter size were available for 832 females (2712 litters, with a mean litter size of 7.4 piglets and standard deviation of 2.3) that were born across all cohorts (i.e., from 0 to 23), so that ΔID could be calculated by including ${F}_{PED}$ as a covariate in an animal model. The detailed statistical model used is given by Saura et al. [14]. Estimates of ΔID from molecular measures of $F$ were also obtained for 103 females (250 litters, with mean litter size of 7.1 piglets and standard deviation of 2.4) that were genotyped in cohorts 17–23 [14].

We carried out a gene dropping simulation of the whole known pedigree and ascribed to the individuals of the initial cohort the simulated genotypic data from the large initial population of 500 individuals, as described before. The pedigree was run 2000 times, starting from random samples from the large initial population to generate the genotypic values for the individuals of the initial cohort (cohort 0). The length of the genome, the number of chromosomes and the recombination rate assumed between nucleotides were the same as those detailed in the previous sections. Simulated offspring were obtained for each mating following the pedigree in the absence of selection and mutation. The different measures of $F$ were obtained for all individuals across cohorts. The mean and variance of the different estimators of inbreeding and the correlations between them were calculated for the last cohort (23), either assuming the allele frequencies of cohort 17 (considered the base population to which $F$ estimates refer to), assuming the frequencies of the current generation (cohort 23), or assuming frequencies equal to 0.5. Finally, using the available data for litter size from 103 females (born in cohorts 17–23), ΔID was obtained for each molecular $F$ estimator using the simulated genotypes. The simulation results were compared with the empirical estimates.

### Molecular measures of the inbreeding coefficient

The following estimators of the inbreeding coefficient were calculated based on the SNP data:

$${F}_{VR1}=\frac{{\sum }_{k=1}^{S}{\left({x}_{k}-2{p}_{k}\right)}^{2}}{{\sum }_{k=1}^{S}2{p}_{k}\left(1-{p}_{k}\right)}-1,$$

where $S$ is the total number of markers, ${x}_{k}$ is the number of minor alleles of marker $k$ (i.e., 0, 1 or 2 copies), and ${p}_{k}$ is the frequency (initial, current or 0.5) of the minor allele. This estimator was proposed by VanRaden [19] and it is based on the variance of additive genetic values.

$${F}_{VR2}=\frac{1}{S}\sum_{k=1}^{S}\left(\frac{{\left({x}_{k}-2{p}_{k}\right)}^{2}}{2{p}_{k}\left(1-{p}_{k}\right)}-1\right),$$

a metric proposed by Amin et al. [67], as cited by VanRaden [19]. It only differs from ${F}_{VR1}$ in that the weighting by the variance of allele frequencies is done for each SNP rather than by the summation of variances for all SNPs, so that it gives a larger weight to rare alleles.

$${F}_{YA2}=\frac{1}{S}\sum_{k=1}^{S}\frac{{x}_{k}^{2}-\left(1+2{p}_{k}\right){x}_{k}+2{p}_{k}^{2}}{2{p}_{k}\left(1-{p}_{k}\right)},$$

which is a coefficient based on the correlation between uniting gametes [20], such that homozygous genotypes are weighted by the inverse of their allele frequencies, and it is expected to show lower sampling variance than other estimators [36].

$${F}_{YA1}=\frac{{\sum }_{k=1}^{S}{x}_{k}^{2}-\left(1+2{p}_{k}\right){x}_{k}+2{p}_{k}^{2}}{{\sum }_{k=1}^{S}2{p}_{k}\left(1-{p}_{k}\right)},$$

an analogous estimator to that of Yang et al. [20] (${F}_{YA2}$) but considering the summation of terms separately in the numerator and denominator, as recommended by Zhang et al. [34].

$${F}_{LH1}=1-\frac{{\sum }_{k=1}^{S}{x}_{k}\left(2-{x}_{k}\right)}{{\sum }_{k=1}^{S}2{p}_{k}\left(1-{p}_{k}\right)},$$

the metric that was initially proposed by Li and Horvitz [16], which gives the deviation of the observed frequency of homozygotes from the expected values under Hardy–Weinberg equilibrium when current allele frequencies are considered.

$${F}_{LH2}=1-\frac{1}{S}\sum_{k=1}^{S}\left(\frac{{x}_{k}\left(2-{x}_{k}\right)}{2{p}_{k}\left(1-{p}_{k}\right)}\right),$$

for which the different weighting in relation to *F*_*LH*1_ is analogous to that between ${F}_{VR2}$ and ${F}_{VR1}$, and between ${F}_{YA2}$ and ${F}_{YA1}$.

$${F}_{q05},$$

which is obtained by setting ${p}_{k}$ = 0.5 for any of the previous estimators.

$${F}_{HOM}=1-\frac{1}{S}{\sum }_{k=1}^{S}{x}_{k}\left(2-{x}_{k}\right),$$

which is the average number of homozygous SNPs, which has a correlation of 1 with ${F}_{LH1}$ and ${F}_{q05}$.

The above estimators may receive different names in different studies and a summary of this nomenclature is in Table 1 of Villanueva et al. [12]. Here, we used a terminology, as simple as possible, by identifying the abbreviation for the authors who originally proposed the estimators (*VR*, *YA*, *LH*) plus a suffix 1 to indicate that the measure is obtained from a ratio of averages over loci (*VR*1, *YA*1, *LH*1) and a suffix 2 when the same estimator is obtained from the average of the ratios (*VR*2, *YA*2, *LH*2). This may simplify the existing “Babel tower” [44] of nomenclature.

The estimators are based either on genetic drift derivations (${F}_{VR1-2}$ and ${F}_{YA1-2}$) or on homozygosity derivations (${F}_{LH1-2}$ and ${F}_{HOM}$) and the equivalence between both approaches was already demonstrated by Cockerham [68]. A summary of the relationships between the estimators is given in the Supplementary Appendix of Caballero et al. [32], and a derivation of ${F}_{YA2}$ from the correlation between uniting gametes is given in the Appendix of this paper.

The measures ${F}_{VR2}$, ${F}_{YA2}$, ${F}_{LH1}$ and ${F}_{LH2}$ can readily be obtained by the software PLINK and GCTA (${F}^{I}$, ${F}^{III}$, $F$ and ${F}^{II}$, respectively), while ${F}_{VR1}$ can be obtained from the diagonal of the genomic relationship matrix obtained by GCTA with the option-make-grm-alg 1.

Note that, for these molecular measures to provide estimates of ${F}_{IBD}$ with reference to a given base population, the calculations must be done using the allele frequencies (${p}_{k}$) of all SNPs that segregate in the base population, rather than of only SNPs that segregate in the generation for which the estimate of inbreeding is computed. Estimates ${F}_{HOM}$ and the coefficient obtained assuming frequencies equal to 0.5 (${F}_{q05}$) were obtained using only SNPs that are segregating in the current generation.

Finally, estimates of the inbreeding coefficient based on ROH (${F}_{ROH}$) were obtained as:

$${F}_{ROH}= \frac{\sum {L}_{ROH}}{L},$$

where $\sum {L}_{ROH}$ is the sum of the lengths of all ROH across the genome of the individual, and $L$ is the genome length [25]. We used the software PLINK [59, 60] with default options, i.e., a minimum of 100 SNPs per ROH (30 SNPs in the simulated pig population to match the conditions assumed by Saura et al. [14]), at least 1 SNP per 50 kb (100 kb in the simulated pig population), a scanning window of 50 SNPs, and two SNPs in the same ROH no more than 1000 kb apart. We considered ROH lengths longer than 1 Mb (${F}_{ROH-1}$) or 5 Mb (${F}_{ROH-5}$).

## Results

### Comparison of estimators of inbreeding and inbreeding depression

#### Default model

**Fig. 1:**
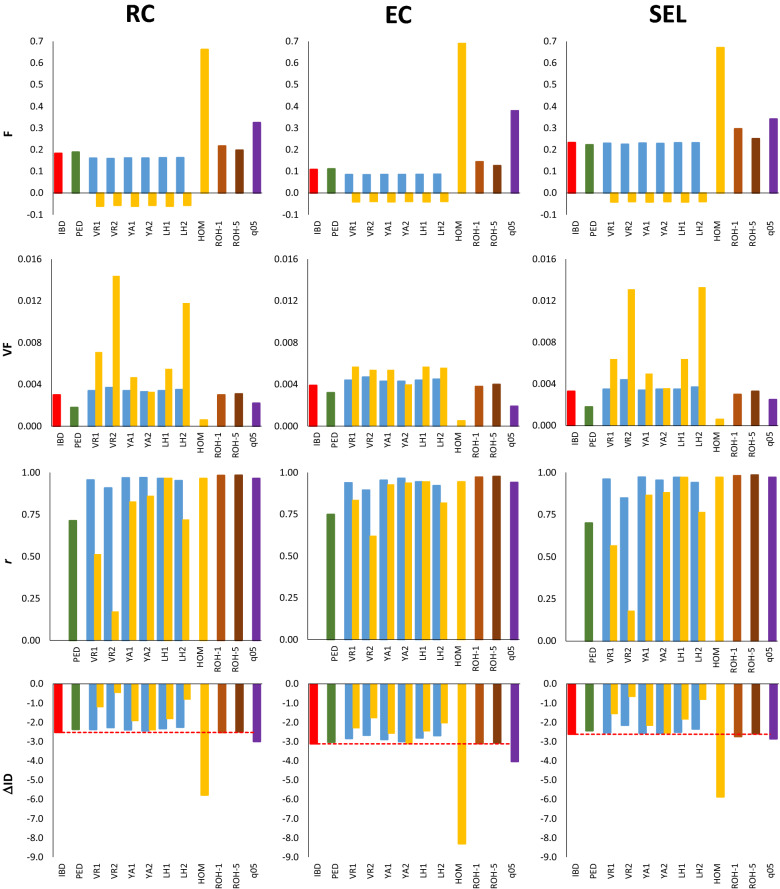
Estimates of inbreeding based on alternative measures, correlation among them, and rate of inbreeding depression for fitness obtained for a population of *N* = 20 breeding individuals maintained for 10 discrete generations assuming random mating and random contributions from parents to progeny (RC), equalization of contributions from parents to progeny (EC), and artificial selection for a neutral quantitative trait (SEL). Mean (*F*) and variance (*VF*) of inbreeding coefficients at generation 10, correlation between estimated inbreeding coefficients and those obtained from IBD measures (*r*), and mean values of the rate of inbreeding depression (ΔID). Bars refer to true IBD values (*F*_*IBD*_; horizontal red line), and estimated from pedigree records (*F*_*PED*_) and from different marker frequency-based measures (*F*_*VR*1_, *F*_*VR*2_, *F*_*YA*1_, *F*_*YA*2_, *F*_*LH*1_, *F*_*LH*2_, *F*_*HOM*_; see text for definitions) assuming the frequencies of the base generation (blue bars), those of the current generation (yellow bars) or a constant frequency of 0.5 (*F*_*q*05_; purple bars). Estimates from runs of homozygosity are shown for fragments larger than 1 Mb (*F*_*ROH*-1_) or 5 Mb (*F*_*ROH*-5_). Only subscripts of estimators are shown for the sake of clarity

Figure 1 shows the comparison of the different estimators of the inbreeding coefficient and ${F}_{IBD}$ values for a population size of $N$ = 20 individuals run for $t$ = 10 generations under the three scenarios: no selection with random contributions (RC), equalization of contributions (EC), and artificial selection (SEL). The two upper rows of the graphs show the averages and variances of the different measures of inbreeding, as well as estimates based on ROH. Note that when frequencies equal to 0.5 are used, all marker-based estimators are equivalent and a single purple bar is represented for all of them. Most of the patterns were very similar for the three schemes, with only some small differences between them.

The average ${F}_{IBD}$ under EC was about one half of that under random contributions, and that under artificial selection was slightly larger than that without selection, as expected (Fig. 1, first row). The average ${F}_{IBD}$ was well estimated by ${F}_{PED}$, and also by the five marker-based inbreeding coefficients when allele frequencies of the base population are known (blue bars), with a slight underestimation due to the excess of heterozygotes expected for markers in finite populations_._ When allele frequencies in the current generation were used instead (yellow bars), all estimators gave the expected average deviation from Hardy–Weinberg proportions (values slightly below zero, expected to be around − 1/2*N*). Estimates from direct average homozygosity of SNPs (${F}_{HOM}$) gave large overestimations of ${F}_{IBD}$, as expected. The average values of ${F}_{ROH}$ were slight overestimations of ${F}_{IBD}$, particularly when including shorter fragments (${F}_{ROH-1}$). Using allele frequencies equal to 0.5 when computing the marker-based estimators (${F}_{q05}$, purple bars) gave also substantial overestimations of ${F}_{IBD}$.

The variances between individual estimates of inbreeding were substantially smaller for ${F}_{PED}$, ${F}_{HOM}$ and ${F}_{q05}$ than for ${F}_{IBD}$ (Fig. 1, second row, *VF*). When the initial allele frequencies were known (blue bars), variances of molecular estimators of inbreeding were very similar to the variance of ${F}_{IBD}$, except that for ${F}_{VR2}$ (particularly for the SEL scheme), which had the largest variance. When allele frequencies of the current generation were used (yellow bars), the variances for molecular estimators of inbreeding increased substantially, although not so much in the EC scenario. The estimator that had the closest variance to that of ${F}_{IBD}$ was ${F}_{YA2}$. The variances of ${F}_{ROH}$ were also close to those of ${F}_{IBD}$.

The third row of the graphs in Fig. 1 shows the correlation between the estimated and true ${F}_{IBD}$ values. When the initial allele frequencies were known (blue bars), all molecular estimators had a very high correlation with ${F}_{IBD}$, except for ${F}_{VR2}$. The ${F}_{ROH}$ estimators also had a very high correlation with ${F}_{IBD}$, while the ${F}_{PED}$ estimator had the lowest correlation with ${F}_{IBD}$. When using allele frequencies in the current generation (yellow bars), ${F}_{LH1}$, ${F}_{HOM},$ and ${F}_{q05}$ (which have a correlation of 1 among them) generally showed the highest correlations with ${F}_{IBD}$.

The fourth row of the graphs in Fig. 1 shows estimates of the inbreeding depression rate (ΔID) compared to the estimate based on ${F}_{IBD}$ (horizontal dotted line). When base population frequencies were known (blue bars), all measures of inbreeding provided estimates of inbreeding depression that were close to those obtained with ${F}_{IBD}$, with estimates based on ${F}_{VR2}$ and ${F}_{LH2}$ slightly biased downwards, particularly in the SEL scenario. Estimates of inbreeding depression based on ${F}_{PED}$ and ${F}_{q05}$ were also rather accurate but ${F}_{q05}$ provided a substantial overestimation of inbreeding depression under the EC scenario. When frequencies in the current generation were used (yellow bars), ${F}_{YA2}$ gave the most accurate estimates of inbreeding depression for the three scenarios, while ${F}_{HOM}$ resulted in very large overestimations and ${F}_{VR2}$ and ${F}_{LH2}$ in the largest underestimations. Estimates of ΔID based on ${F}_{ROH}$ were very close to those from ${F}_{IBD}$. Statistical tests to compare the mean difference between the estimates of ΔID based on ${F}_{IBD}$ with those based on the different marker-based estimators are shown in Additional file 4: Table S2. All differences were significantly greater than 0 except for estimates based on ${F}_{ROH-1}$ in the RC and EC scenarios, based on ${F}_{PED}$ in the EC scenario, and based on ${F}_{YA2}$ when using the current frequencies in the EC scenario.

Additional file 5: Fig. S3 shows results analogous to those in Fig. 1 but after 20 instead of 10 generations, so that the average inbreeding coefficient was doubled, while Additional file 6: Fig. S4 shows results for $N$ = 100 run after 50 generations. In general, the results in these two figures were similar to those presented in Fig. 1, although there were some relevant differences. With $N$ = 20 after 20 generations [see Additional file 5: Fig. S3], using base population allele frequencies equal to 0.5 (purple bars) resulted in underestimation of average inbreeding and ΔID for the RC and SEL schemes. With $N$ = 100 after 50 generations [see Additional file 6: Fig. S4], the increase of $F$ by selection (scheme SEL) was more evident than in the previous cases (with some overestimation of the molecular estimates), and the estimates of ${F}_{PED}$ were more clearly biased downwards.

Additional file 7: Table S3 gives the minimum and maximum values of individual inbreeding coefficients for the simulations presented in Fig. 1 and in Additional file 5: Fig. S3 and Additional file 6: Fig. S4. As expected, ${F}_{IBD}$, ${F}_{PED}$, ${F}_{HOM}$ and ${F}_{ROH}$, which are probabilities, ranged from 0 to 1, while ${F}_{VR1-2}$, ${F}_{YA1-2}$ and ${F}_{LH1-2}$, which are correlations or deviations from Hardy–Weinberg proportions, ranged from – 1 to 1. However, some values of ${F}_{VR2}$ were greater than 1 and some values of ${F}_{LH2}$ were smaller than – 1.

The power of the different measures of inbreeding to detect inbreeding depression is shown in Additional file 8: Fig. S5 for the scenarios considered in Fig. 1 and in Additional file 5: Fig. S3 and Additional file 6: Fig. S4. When allele frequencies of the base population were known, all estimators (as well as ${F}_{ROH}$ and ${F}_{q05}$) resulted in approximately the same power as ${F}_{IBD}$, although some differences were observed between estimators in the case with $N$ = 100 [see Additional file 8: Fig. S5c]. When allele frequencies in the base population were unknown, the most powerful estimator was generally ${F}_{YA2}$ (or ${F}_{YA1}$, with a slightly higher power in some occasions). The ${F}_{VR2}$ and ${F}_{LH2}$ estimators generally showed the lowest power. The power of ${F}_{PED}$ to detect inbreeding depression was usually lower than that of marker-based estimators.

Correlations between individual values for all estimators of inbreeding are shown in Additional file 9: Fig. S6. When allele frequencies in the base population were known [see Additional file 9: Fig. S6a–c], pairwise correlations between marker-based estimators were generally very large (≥ 0.9), with the correlation between ${F}_{VR2}$ and ${F}_{LH1-2}$ being somewhat lower. Correlations of ${F}_{PED}$ with the marker-based estimators were lower than correlations among the latter estimators. When the allele frequencies in the current generation were used, correlations were generally lower than when frequencies in the base population were used but still moderately high [see Additional file 9: Fig. S6d–f]. Some correlations with ${F}_{VR2}$ or ${F}_{LH2}$ were close to zero or negative. The ${F}_{ROH}$ estimator was highly correlated with the other estimators except with ${F}_{VR2}$, particularly in the SEL scenario.

#### Alternative models

In order to assess the robustness of the previous results, we also ran scenario RC under alternative conditions. Additional file 10: Fig. S7a shows results for the scenario with $N$ = 20 after 10 generations but with double the density of SNPs than in Fig. 1 (see Table 1). Additional file 10: Fig. S7b shows results for the same scenario except that the effect of deleterious mutations was 1/4 of that used for the default model. In addition, Additional file 10: Fig. S7c presents results for $N$ = 100, as in Additional file 6: Fig. S4, but after 100 generations instead of 50. In general, results for these alternative models were very similar to those of the default model, confirming the main results. Finally, Additional file 10: Fig. S7d shows that results were similar to those of Additional file 5: Fig. S3 with $N$ = 20 after 20 generations but with the base population set up at generation 10 rather than generation 0. The only relevant difference was that ${F}_{ROH}$ overestimated ${F}_{IBD}$ and ΔID, which is expected because ${F}_{IBD}$ is relative to generation 10 whereas ${F}_{ROH}$ provides values of inbreeding relative to more ancestral generations.

**Fig. 2:**
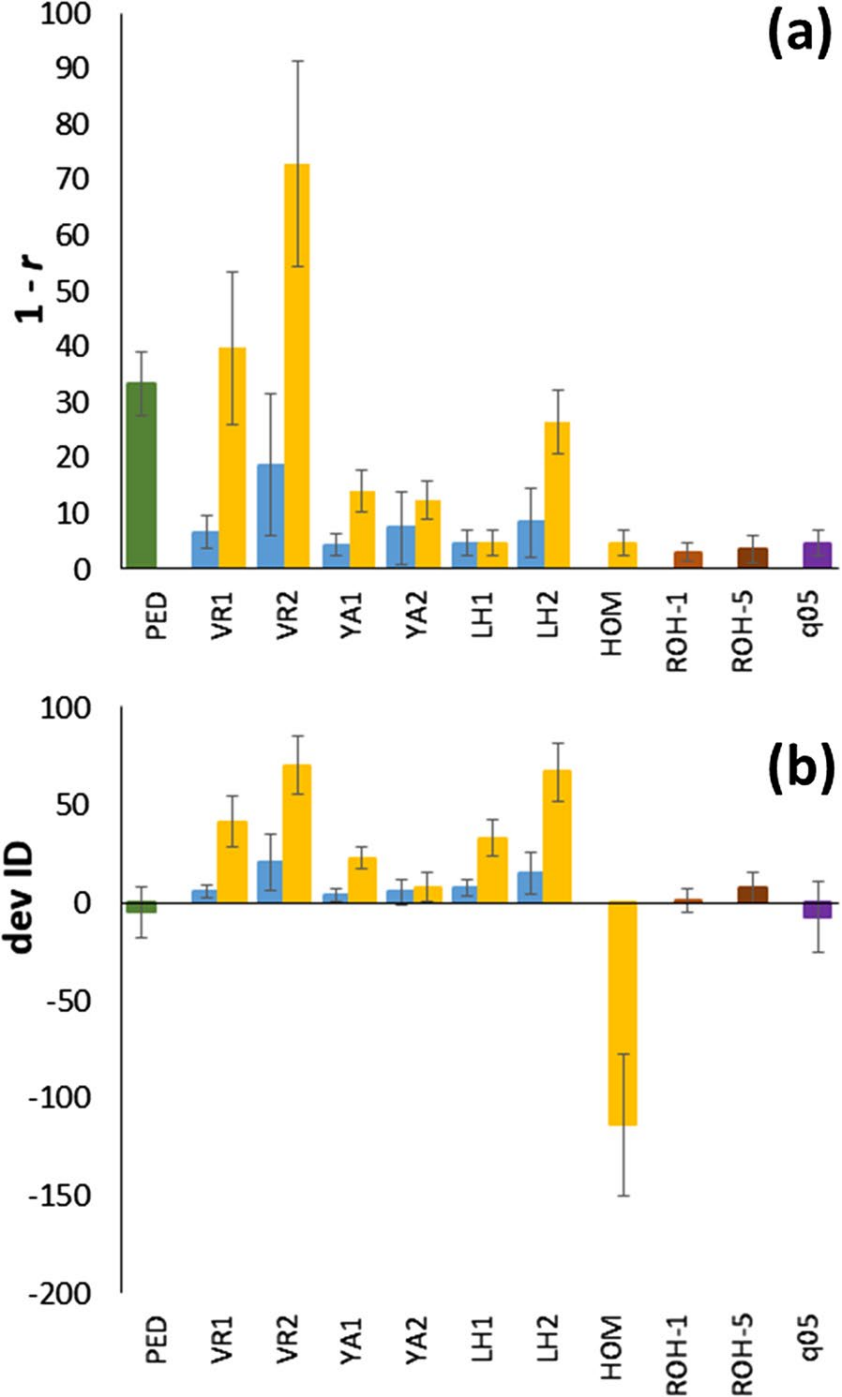
**a** Correlation (*r*), expressed as 1–*r* (in percentage), of the different estimates of inbreeding with true IBD inbreeding, and **b** Average difference between estimates of the rate of inbreeding depression (ΔID) and those obtained from true IBD inbreeding. All results in the study from Fig. 1, Additional file 5: Fig. S3, Additional file 6: Fig. S4 and Additional file 10: Fig. S7a–c were averaged (intervals denote 1 standard deviation). The estimates refer to pedigree records (*F*_*PED*_; green bars) and to different marker frequency-based measures (*F*_*VR*1_, *F*_*VR*2_, *F*_*YA*1_, *F*_*YA*2_, *F*_*LH*1_, *F*_*LH*2_, *F*_*q*05_; see text for definitions), assuming the frequencies of the base generation (blue bars), those of the current generation (yellow bars), or a constant frequency of 0.5 (purple bars). Estimates from runs of homozygosity are shown for fragments larger than 1 Mb (*F*_*ROH*-1_) or 5 Mb (*F*_*ROH*-5_). Only subscripts of estimators are shown for a better view

Because the general patterns observed for the different scenarios (RC, EC, SEL, and different *N* values) were rather similar, Fig. 2 shows a summary of the main global findings by pooling the results for all breeding scenarios and simulations included in Fig. 1, Additional file 5: Fig. S3, Additional file 6: Fig. S4, and Additional file 10: Fig. S7a–c. Average correlations of the different estimates of the inbreeding coefficient with ${F}_{IBD}$ are presented as one minus the correlation (in percentage), such that lower values imply a greater accuracy of estimating ${F}_{IBD}$ (Fig. 2a). When the allele frequencies of the base population were known (blue bars), all molecular estimators of inbreeding showed a good accuracy for estimating ${F}_{IBD}$, although ${F}_{VR2}$ was slightly less accurate. When allele frequencies in the current generation were used (yellow bars), ${F}_{LH1}$ (or ${F}_{HOM}$) gave the most accurate estimation of ${F}_{IBD}$. Good estimates of ${F}_{IBD}$ were also obtained when the frequencies of the base population were assumed to be 0.5 (purple bar) or when ${F}_{ROH}$ estimates were considered (brown bars).

Figure 2b gives the average difference between the estimate of ΔID based on the different estimators compared to the estimate based on ${F}_{IBD}$ for all scenarios. Positive values imply underestimation of ΔID and negative values imply overestimation. Both ${F}_{PED}$ and all molecular estimators, except ${F}_{VR2}$ and ${F}_{LH2}$, gave relatively good average estimates of ΔID when allele frequencies in the base population were known or when a frequency of 0.5 was assumed. If allele frequencies in the base population were not known, the best estimator of ΔID was ${F}_{YA2}$, although ${F}_{ROH}$ also provided little biased estimates of ΔID. The ${F}_{HOM}$ estimator showed large overestimations of ΔID.

### Comparison between simulated and empirical results in an Iberian pig population

**Fig. 3:**
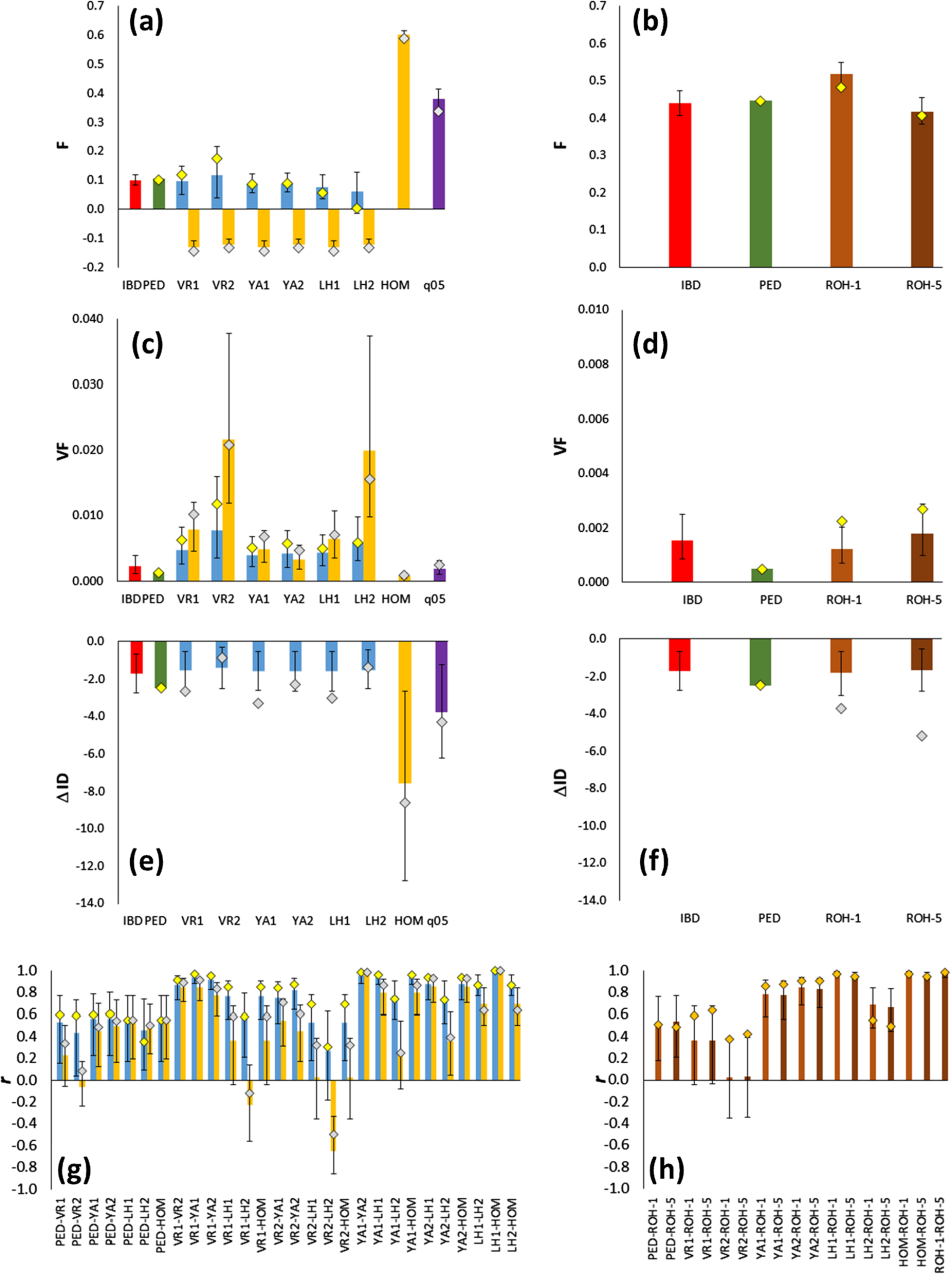
Simulated (bars) and empirical (diamonds) estimates of inbreeding for the Guadyerbas pig population. Mean (*F*) and variance (*VF*) of simulated inbreeding coefficients in cohort 23, obtained from true IBD values (*F*_*IBD*_), and estimated from pedigree records (*F*_*PED*_) and from markers (*F*_*VR*1_, *F*_*VR*2_, *F*_*YA*1_, *F*_*YA*2_, *F*_*LH*1_, *F*_*LH*2_, *F*_*ROH*_; see text for definitions) assuming the frequencies of the base generation (cohort 17 in **a** and **c** and cohort 0 in **b** and **d**; blue bars and yellow diamonds), those of the current generation (cohort 23; yellow bars and grey diamonds) or a constant frequency of 0.5 (purple bars). **e** and **f** Estimates of the inbreeding depression rate (ΔID) for litter size from 103 females born in cohorts 17–23. The estimate from *F*_*PED*_ was obtained from data of 832 females born in all cohorts (0–23). **g** and **h** Correlations among simulated inbreeding coefficients in cohort 23. The intervals shown denote 95% of the variation across simulated replicates in order to see if the observed values are within the expected simulated values. Only subscripts of estimators are shown for a better view

Figure 3 presents simulated average estimates of $F$, their variance, and correlations between different measures of $F$ using individuals in the last generation (cohort 23). Estimates were obtained assuming allele frequencies from cohort 17, the current generation (cohort 23), or equal frequencies of 0.5. The figure also shows simulated values of ΔID (Fig. 3e, f). The simulation results generally agreed with those obtained for the discrete generation simulations, although results here referred to a single simulated pedigree (the empirical one). The empirical results were usually within the 95% of the distribution of simulation results across replicates, concurring with some of their major findings. For example, estimates of the average inbreeding coefficient based on ${F}_{VR2}$ and ${F}_{LH2}$ when the allele frequencies in the base population were known ($F$, yellow diamonds) had the largest differences with ${F}_{IBD}$ and the largest variances in $F$ values (Fig. 3a, c). In addition, estimates from these estimators were highly negatively correlated (Fig. 3g), as predicted by the simulations. When base population frequencies were assumed to be equal to 0.5 (${F}_{q05}$), substantially overestimated ΔID based on ${F}_{IBD}$ (Fig. 3e) were obtained, in agreement with the overestimations found in the scenario EC in the simulations (Fig. 1, Additional file 5: Fig. S3 and Additional file 6: Fig. S4).

**Fig. 4:**
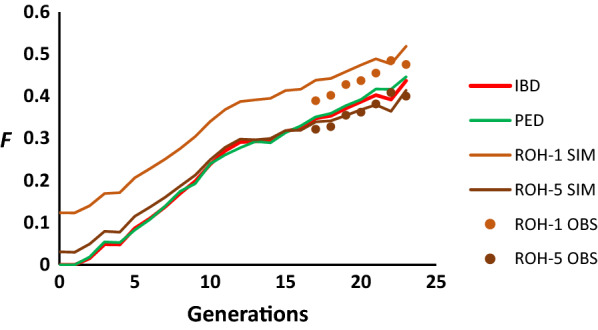
Change in the average simulated and observed inbreeding coefficient across generations for the Guadyerbas pig population. Lines refer to average simulated IBD values (*F*_*IBD*_), estimated from pedigree records (*F*_*PED*_) and from simulated (SIM) and observed (OBS; circles) runs of homozygosity considering fragments larger than 1 Mb (*F*_*ROH*-1_) or 5 Mb (*F*_*ROH*-5_). Only subscripts of estimators are shown for clarity

Because ROH estimates of inbreeding consider more ancient inbreeding (i.e., inbreeding relative to a more ancient generation) than that of cohort 17, Fig. 3b, d, f, h shows simulation and observed results assuming that the base population is the initial cohort 0. The average inbreeding coefficient based on ${F}_{ROH-5}$ was very close to that based on ${F}_{IBD}$, while that based on ${F}_{ROH-1}$ was higher, as it may also consider more ancient inbreeding. This is well illustrated in Fig. 4, which shows the simulated and observed average inbreeding coefficients based on ${F}_{IBD}$, ${F}_{PED}$, ${F}_{ROH-1}$, and ${F}_{ROH-5}$ across generations. Estimates of inbreeding based on ${F}_{ROH-5}$ were very close to ${F}_{IBD}$ and ${F}_{PED}$ values. For the simulations, the average number of ROH per individual considering all individuals between cohorts 17 and 23 was about 86, with an average length of 10 Mb for ROH > 1 Mb, and about 60 with an average length of 13 Mb for ROH > 5 Mb. These values are similar to those found in the real data: an average of 77.7 ROH per individual with a mean length of 9.99 Mb for ROH > 1 Mb, and 47.9 ROH with a mean length of 14.04 Mb for ROH > 5 Mb. There was, however, some discrepancy between simulated and empirical estimates of ΔID based on ${F}_{ROH}$, with the empirical estimates of the latter being higher (Fig. 4f).

## Discussion

Proper estimation of the inbreeding coefficient of individuals is necessary to estimate inbreeding depression and effective population size in domestic and wild populations. The possibility of obtaining data from large numbers of SNPs for an increasing number of species allows the estimation of genomic inbreeding in the absence of pedigree records or as a complement to them. Here, we carried out computer simulations to compare some of the most popular marker-based estimators of $F$ that are readily obtained from widely-used software such as PLINK and GCTA under different scenarios, including equalization of parental contributions, and artificial and natural selection. Our results show that, all estimators generally had high correlations with ${F}_{IBD}$ and precise estimates of ΔID when the allele frequencies in the base population are known, with ${F}_{VR2}$ and ${F}_{LH2}$ having the lowest performance (Fig. 2). When allele frequencies in the base population are not known, *F*_*LH*1_ generally had the largest correlation with ${F}_{IBD}$, and ${F}_{YA2}$ generally gave the most accurate estimates of ΔID. The ${F}_{VR2}$ and ${F}_{LH2}$ estimators had very low correlations with ${F}_{IBD}$ and low accuracies to estimate ΔID in most scenarios. The assumption of allele frequencies of 0.5 in the calculation of the estimators (${F}_{q05}$) resulted in high correlations with ${F}_{IBD}$ and relatively good estimates of ΔID, except in the EC scenario, for which a substantial overestimation was found. Estimates of ΔID obtained with simple homozygosity measures (${F}_{HOM}$) resulted in large overestimations of ΔID, although ${F}_{HOM}$ has a correlation of 1 with ${F}_{LH1}$; however, its variance is much lower than that of ${F}_{IBD}$ (Fig. 1), resulting in estimates of ΔID based on ${F}_{HOM}$ to be biased upwards. This agrees with previous studies that showed that estimates of ΔID based on homozygosity measures of $F$ are generally larger than those based on other estimators of $F$ [37, 49]. Estimates of inbreeding based on ROH gave precise estimates of ${F}_{IBD}$ and ΔID, as also shown by previous studies [9, 27–30, 47]. Finally, estimates of $F$ based on pedigrees (${F}_{PED}$) were less correlated with ${F}_{IBD}$ than molecular estimates, but provided estimates of ΔID with little bias. Our results for the pedigreed pig population showed generally good agreement between simulated and empirical results, supporting the above findings.

### Estimation of the inbreeding coefficient

When allele frequencies for a base population of reference that consists of noninbred and unrelated individuals are known, all marker frequency-based estimators are expected to give almost unbiased estimates of ${F}_{IBD}$ [19, 22, 31, 32], as observed in our simulation results (Fig. 1). The slightly lower average inbreeding coefficients based on molecular estimates compared to ${F}_{IBD}$ (Fig. 1) is explained by the negative excess of heterozygotes expected for finite population sizes, which amounts approximately to − 1/2*N* [69, 70]. Note that, as the use of genomics becomes more common, it will be more frequent to have several previous generations genotyped and, therefore, to have information available for a base initial population of reference. Allele frequencies for a base population can also be estimated. Gengler et al. [33] and, more recently, Aldrige et al. [71] have proposed practical methods to calculate gene content using a linear regression. However, these methods are not feasible for deep pedigrees, and it is standard practice to use allele frequencies for the current genotyped population because of the ease of computation. Even if base population frequencies are available, the correlation with ${F}_{IBD}$ is not the same for all estimators. The ${F}_{LH1}$, ${F}_{VR1},$ and ${F}_{YA1-2}$ estimators provided accurate predictions, but ${F}_{VR2}$ and ${F}_{LH2}$ did not. The latter (called ${F}^{II}$ in the software PLINK and GCTA) is almost never used in practice. However, ${F}_{VR2}$ (${F}^{I}$ in PLINK and GCTA) has been considered on many occasions [12, 41, 43, 44, 50, 58]. Our results argue against the use of both these estimators, as they showed the lowest correlations with ${F}_{IBD}$ and the most biased estimates of ΔID in the majority of scenarios, as summarized in Fig. 2.

When allele frequencies in the base population are not known, and those of the current population are used instead, which is the most frequent situation, the most accurate marker-frequency-based estimator of ${F}_{IBD}$ was ${F}_{LH1}$ (or ${F}_{HOM}$ or ${F}_{q05}$, which both had a correlation of 1 with ${F}_{LH1}$). ${F}_{LH1}$ had the highest correlation with total homozygosity [32], and it is also the most useful to indicate if genetic diversity has been lost or gained in specific regions of the genome from the past generations to the current one [12]. For example, an increase in the value of local ${F}_{LH1}$ indicates a loss in heterozygosity, while a decrease indicates a gain in heterozygosity. In contrast, ${F}_{VR1}$, ${F}_{VR2},$ and ${F}_{YA2}$ do not provide useful information of whether heterozygosity has declined or increased in genomic regions [12].

Estimates of the inbreeding coefficient based on ROH were very accurate estimators of ${F}_{IBD},$ although they depended on the length of the fragments considered. For the pig data, Fig. 4 shows that estimates of ${F}_{ROH-5}$ were very close to the expected values of ${F}_{IBD}$ and ${F}_{PED}$ relative to the initial cohort, while ${F}_{ROH-1}$ provide larger estimates of inbreeding. Because the average rate of recombination for pigs is about 0.8 cM per Mb, ${F}_{ROH-5}$ values refer to events of inbreeding that occurred since about 12.5 generations ago [9], which probably includes most of the inbreeding that occurred since the foundation of the strain. However, ${F}_{ROH-1}$ refers to events of inbreeding that occurred further in the past (on average about 62.5 generations), which includes inbreeding that occurred before foundation of the strain, and explains why the mean ${F}_{ROH-1}$ in cohort 0 was greater than 0 (0.1).

Estimates of inbreeding based on current allele frequencies had very variable correlations with each other and with pedigree-based estimates of inbreeding. For example, the non-exhaustive compilation of correlations in Table 2 shows that the correlation of the estimates of inbreeding based on homozygosity (${F}_{LH1}$ or ${F}_{HOM}$) with ${F}_{PED}$ ranged from 0.1 to 0.9, with an average of about 0.5. Correlations of other marker-based estimators with ${F}_{PED}$ and with each other were also rather variable, with some negative values, usually involving ${F}_{VR2}$. These empirical results from Table 2 agree with our results, which also showed the lowest correlation between ${F}_{PED}$ and ${F}_{VR2}$, both in the simulations [see Additional file 9: Fig. S6] and in the pig data (Fig. 3). Correlations between molecular estimators of $F$ were generally high, except for some correlations that involved ${F}_{VR2}$ particularly with ${F}_{LH1}$ and ${F}_{LH2}$, which were often low or negative (Table 2). This is also evident from our simulations results and the pig data ([see Additional file 9: Fig. S6] and Fig. 3), as well as from simulation results by Zhang et al. [34], where correlations of ${F}_{VR2}$ with ${F}_{PED}$, ${F}_{LH1}$, and ${F}_{YA1}$ were negative. Moreover, minima and maxima of individual inbreeding coefficients were within the expected ranges in our study [see Additional file 7: Table S3]), except for some values higher than 1 or lower than – 1 for ${F}_{VR2}$ and ${F}_{LH2}$, which are the estimators with the poorest performances. Therefore, the different estimators allow for a wide range of possible values of *F*, which likely depends on many factors such as the species, mating system, family structure, density and quality of markers, population size, etc. In addition, empirical results suggest that genotype imputation largely increases the variance of the estimators, thus producing substantial overestimates of average inbreeding coefficients and individual values well outside the expected ranges [44].

**Table 2:** Compilation from the Literature of correlations between individual measures of inbreeding from empirical data based on marker-based estimators of inbreeding assuming allele frequencies in the current generation and based on runs of homozygosity (ROH)

	*F*_*VR*1_	*F*_*VR*2_	*F*_*YA2*_	*F*_*LH*1_ or *F*_*HOM*_	*F*_*LH*2_	*F*_*ROH*_
*F*_*PED*_	**0.21** [41, 42, 51, 56, 57]	**− 0.09** [39, 41, 52, 54, 55]	**0.42** [13, 39, 42, 49, 55, 57]	**0.50** [13, 14, 39, 41, 42, 49, 51, 52]	**0.32** [55]	**0.50** [13, 14, 39, 41, 49, 51]
	0.10–0.39	− 0.26–0.21	0.15–0.72	0.10–0.78		0.07–0.92
*F*_*VR*1_		**0.91** [12, 41, 43, 50]	**0.84** [12, 42, 43, 50, 57]	**0.42** [12, 41–43]		**0.57** [51, 56, 57]
		0.83–0.97	0.71–0.92	0.11–1.0		− 0.13–0.99
*F*_*VR*2_			**0.52** [12, 39, 43, 50, 55]	**− 0.21** [12, 39, 41, 43, 50, 52, 54, 55]	**− 0.64** [55]	**0.01** [39, 41, 52, 54, 55, 58]
			0.26–0.75	− 0.95–0.36		− 0.26–0.22
*F*_*YA2*_				**0.64** [12, 13, 39, 42, 43, 49, 50, 55]	**− 0.04** [55]	**0.78** [13, 39, 49, 55, 57, 58]
				− 0.24–0.92		0.50–0.95
*F*_*LH*1_ or *F*_*HOM*_					**0.72** [55]	**0.83** [13, 14, 39, 41, 49, 51–53]
						0.31–0.99
*F*_*LH*2_						**0.66** [55]

The compilation is not intended to be exhaustive, but to give a general picture of the empirical correlations found in different studies with several marker-based estimators of inbreeding. *F*_*VR*1_ and *F*_*VR*2_: Estimators described by VanRaden [19]. *F*_*YA*1_ and *F*_*YA*2_: Estimator proposed by Yang et al. [20] and modified by Zhang et al. [34]. *F*_*LH*1_ and *F*_*LH*2_: Estimators derived by Li and Horvitz [16]. *F*_*HOM*_: Average marker homozygosity. *F*_*ROH*_: Estimator from Runs of Homozygosity. Averages in each cell in bold face and the range of values are shown below it

Villanueva et al. ([12]; Guadyerbas pig strain). Pryce et al. ([13]; Holstein and Jersey dairy cattle). Saura et al. ([14]; Guadyerbas pig strain). Zhang et al. ([39], Holstein, Jersey and Danish Red Cattle breeds, sequence data). Brito et al. ([41]; Goat breeds). Alemu et al. ([42]; Dutch Holstein cattle). Morales-González et al. ([43]; Turbot). Bérénos et al. ([49]; Soay sheep). Solé et al. ([50]; Belgian Blue cattle). Yoshida et al. ([51], pure lines of farmed coho salmon). Rodríguez-Ramilo et al. ([52]; Rabbits). Antonios et al. ([53]; Dairy Sheep). Shi et al. ([54]; Pigs). Schiavo et al. ([55]; seven Pig breeds). Adams et al. ([56]; three Turkey lines). Polak et al. ([57]; Polish cold-blooded horses). Nosrati et al. ([58]; Average for 68 sheep populations). Saura et al. ([66]; Iberian pigs)

### Estimation of the rate of inbreeding depression

Regarding estimation of ΔID when current allele frequencies are used, the most accurate molecular marker frequency-based estimator of inbreeding appears to be ${F}_{YA2}$. This is the estimator that results in a variance of *F* values that is closest to that of ${F}_{IBD}$ (see *VF* in Fig. 1, Additional file 5: Fig. S3 and Additional file 6: Fig. S4), and the lowest variance of individual estimates among all molecular estimators, as deduced theoretically by Yang et al. [36] and confirmed by other simulation [32] and empirical [42] results. ${F}_{YA2}$ was also the estimator with the greatest power to detect inbreeding depression [see Additional file 8: Fig. S5]. There has been a number of simulation studies that have investigated the performance of different measures of inbreeding to estimate ΔID when allele frequencies of the current population are assumed [32, 46, 47]. These studies suggest that ${F}_{YA2}$ and ${F}_{ROH}$ generally provide good estimations of ΔID, depending on the population size, while ${F}_{LH1}$ slightly underestimated ΔID and ${F}_{VR2}$ severely underestimated ΔID. In fact, ${F}_{YA2}$ and ${F}_{ROH}$ were highly correlated (Table 2) and previous work has shown that ${F}_{YA2}$ and ${F}_{ROH}$ have approximately the same power to detect inbreeding depression [13, 37], as also confirmed by our simulation results [see Additional file 8: Fig. S5]. It has also been found [32] that ${F}_{YA2}$ has the highest correlation with the phenotypic values of individuals and with the homozygous mutation load [9], as a proxy for fitness.

However, results from previous simulations demonstrate that ${F}_{YA2}$ can substantially overestimate ΔID for relatively small population sizes [32, 47]. For example, an overestimation by about 28% was observed for a population of size of *N* = 500 [32]. This substantial overestimation of ΔID based on ${F}_{YA2}$ was not observed in our results, where ${F}_{YA2}$ was always rather accurate. The reason for this discrepancy is that the previous studies analysed samples that were taken directly from a relatively large population, while we simulated a number of generations with a much smaller $N$ (20 or 100) prior to the generation that was analysed, and mutation was not considered in the simulations. Thus, in our simulations, many rare alleles were likely lost by genetic drift during the period of reduced census size, which may have improved the estimation of the rate of inbreeding depression. In order to check this, we also analysed the case with $N$ = 100 with the RC scheme at generation 0, i.e., immediately after sampling from the large original populations with $N$ = 500. Resulting estimates of ΔID are given in Additional file 11: Fig. S8, which show that, with no minor allele frequency (MAF) filter (red bars), ${F}_{YA2}$ overestimated ΔID by about 28%, ${F}_{LH1}$ underestimated ΔID by about 30%, while ${F}_{ROH}$ produced almost unbiased estimates and ${F}_{VR2}$ and ${F}_{LH2}$ gave large underestimations. These results are in full agreement with those previously reported [32]. However, if these analyses are done when rare alleles were removed by applying a MAF filter of 0.01 or 0.05 to the data, overestimation of ΔID by ${F}_{YA2}$ was reduced to 3% for the 0.01 filter and became an underestimation by 15% for the 0.05 filter [see Additional file 11: Fig. S8]. Thus, part of the overestimation of ΔID that occurred with ${F}_{YA2}$ can be due to the contribution of SNPs with very low frequencies. Estimates of ΔID based on ${F}_{VR1}$, ${F}_{YA1}$*,* and ${F}_{LH1}$, i.e. those based on ratios of averages over loci, were not affected by applying an MAF filter, while those based on ${F}_{VR2}$, ${F}_{LH2}$, and ${F}_{ROH}$ were increased by an increase of the MAF threshold [see Additional file 11: Fig. S8].

The results of our study are concordant with those of Wang [23]. Under the conditions considered in our simulations (genome size of 18 Morgans, number of SNPs > 30,000, population sizes < 100, and number of generations > 20), molecular estimates were more precise estimates of ${F}_{IBD}$ and ΔID than those based on pedigree. Wang [23] considered random mating populations under a model of deleterious mutations on fitness, where the molecular inbreeding metric (${F}_{LH1}$) was calculated using current allele frequencies. We reached similar conclusions (Fig. 2a) for other molecular estimators of inbreeding and scenarios with equalization of contributions and artificial selection. Our results also concur with those of Forutan et al. [48], who compared estimates of inbreeding obtained from pedigree and from molecular data, i.e., ${F}_{VR1}$ and ${F}_{ROH}$, considering random mating and artificially selected populations, and a base population simulated assuming allele frequencies equal to 0.5 or uniformly distributed between 0 and 0.5. They showed that pedigree-based estimates of inbreeding underestimate inbreeding in selected populations, a result that we also observed in our simulations [see Additional file 6: Fig. S4]. This is as expected because selected individuals tend to be more related than the mean of the population [72]. They also found that ${F}_{VR1}$ estimates were close to ${F}_{IBD}$ estimates if the allele frequencies of the base population are known, but assuming a constant allele frequency of 0.5 resulted in overestimation when using ${F}_{VR1}$, although the correlation of these estimates with ${F}_{IBD}$ was close to 1. In fact, high correlations (> 0.6) between inbreeding values obtained based on ${F}_{PED}$ and ${F}_{q05}$ have been found in several empirical studies [35, 37, 48]. This agrees with our results, which also showed that ${F}_{q05}$ generally provided accurate estimates of ΔID (Fig. 2b), except for the EC scheme, where it resulted in overestimation (Fig. 1, Additional file 5: Fig. S3 and Additional file 6: Fig. S4), which was also reflected by the pig data (Fig. 3), a population that was intended to follow an EC protocol.

Pedigree-based inbreeding (${F}_{PED}$) showed rather accurate mean estimates of ${F}_{IBD}$ (see Fig. 1, Additional file 5: Fig. S3 and Additional file 6: Fig. S4), except in the case of artificial selection (see Additional file 6: Fig. S4 and [48]), but had only a moderate correlation with ${F}_{IBD}$ (Fig. 2a), as also found by Wang [23]. Estimates of ΔID by ${F}_{PED}$ were on the whole rather accurate (Fig. 2b), which concurs with the meta-analysis of Doekes et al. [73], who found moderate to high correlations of the estimates of ΔID based on ${F}_{PED}$ with those obtained based on molecular estimators of inbreeding: i.e., about 0.5–0.6 for ${F}_{ROH}$, about 0.6–0.7 for ${F}_{VR2}$ or ${F}_{YA2}$ (increasing to 0.8–0.9 for ${F}_{YA2}$ when base population frequencies of 0.5 were used), and about 0.6–0.7 for ${F}_{LH1}$. According to Wang [23], molecular estimates of inbreeding are more accurate predictors of ${F}_{IBD}$ than pedigree-based estimates when population sizes are smaller than ~ 200 (see his Fig. 3). This agrees with other simulation results assuming small population sizes [28]. However, Wang [23] also showed that for larger population sizes (> 200), the accuracy of pedigree-based estimates of inbreeding increased and became higher than that from molecular estimates of inbreeding. Whereas the accuracy of the molecular estimates of inbreeding was reduced with increasing population size, that from pedigree-based estimates remained invariable with increasing population size (see his Fig. 3). In addition, power to detect inbreeding depression became greater for pedigree-based estimates than for molecular estimates when population sizes were larger than about 500 (see Figure 5 of Wang [23]). Therefore, for large population sizes, under some situations, estimates of inbreeding and ΔID obtained from pedigrees can be as reliable, or even more reliable, than estimates based on molecular markers, although pedigrees for such large populations may only be available in domestic species. In most situations, therefore, estimates of inbreeding and ΔID should be more reliably obtained from molecular markers than from pedigrees.

Our simulations implied that the effective population size (${N}_{e}$) was close to the census size of breeders ($N$), except for the EC scenario, for which ${N}_{e}$ ≈ 2$N$; see e.g., [4], p. 110). Thus, the results referred to scenarios with relatively small ${N}_{e}$ (≤ 100), with average inbreeding coefficients reaching relatively high values ($F$ ≈ 0.1 − 0.4). This is a common situation in many animal breeding populations [74] and populations of conservation concern [75]. Other simulation studies have focused on populations with much larger population sizes (e.g., $N$ > 10,000 individuals) and these have shown that molecular estimators of inbreeding perform also rather well [23, 32, 46].

In order to estimate inbreeding depression for fitness, we assumed a model of partially recessive deleterious mutations with average mutational parameters based on empirical data (see, e.g., [4]; p. 152–161). We also considered an alternative model with a larger number of mutations with much lower effects (Table 1 and Additional file 1: Fig. S1) but this did not affect the main findings of the study. However, we did not consider models of overdominance or epistasis. Overdominance can make some contribution to inbreeding depression but this is generally considered to be minor compared to partial dominance [76–78]. Nevertheless, further studies should be devoted to the investigation of the performance of the molecular estimators of inbreeding under other genetic models.

## Conclusions

If the allele frequencies of the base population are known, all marker frequency-based estimators of inbreeding and inbreeding depression are reasonably highly correlated with ${F}_{IBD}$ and provide accurate estimates of ΔID, except for ${F}_{VR2}$ and ${F}_{LH2}$. If the allele frequencies of the base population are not known, ${F}_{LH1}$ is generally the estimator that is best correlated with ${F}_{IBD}$ and ${F}_{YA2}$ is the best estimator of ΔID. ${F}_{ROH}$ is a very accurate estimator of ${F}_{IBD}$ in most situations, while ${F}_{VR2}$ and ${F}_{LH2}$ perform very poorly in almost all scenarios. Estimates that are obtained assuming a constant frequency of 0.5 (${F}_{q05}$) give highly biased estimates of ${F}_{IBD}$ but are highly correlated with ${F}_{IBD}$ and provide reasonably good estimates of ΔID. Estimates from simple frequencies of homozygous markers (${F}_{HOM}$) cannot be used to estimate ΔID.

### Supplementary Information

**Additional file 1: Figure S1.** Distribution of mutational homozygous effects and dominance coefficients assumed in the simulations. **a** Homozygous effects (*s*) had an exponential distribution with mean effect $\overline{s }$ = 0.1 or 0.025. The number of mutations are scaled by the haploid mutation rate in each model. **b** Dominance coefficients (*h*) were assumed to have an inverse relationship with *s* values and were taken from a uniform distribution between 0 and e^(−*ks*)^, where *k* is a constant needed to get an average value of $\overline{h }$ = 0.2.

**Additional file 2: Table S1.** Number of deleterious loci at generation 0 and those lost, fixed or segregating at generation 20 and their average frequency, for a range of selection coefficients (*s*). The results refer to a simulation with *N* = 20 individuals carried for 20 generations with scheme RC.

**Additional file 3: Figure S2.** Average inbreeding load (*B*), additive (*V*_*A*_) and dominance (*V*_*D*_) variances for fitness for a population of *N* = 20 breeding individuals maintained for 20 discrete generations assuming random mating and random contributions from parents to progeny (RC). The inbreeding load (*B*) measures the cumulative deleterious effect of (partially) recessive mutations that is hidden in large non-inbred populations and is expressed by inbreeding and is quantified in terms of number of lethal equivalents per haploid genome. In the absence of selection, *B* equals the rate of inbreeding depression (ΔID). The values of *B* in the simulations were calculated as the sum over loci of 2*dpq* ([4], p. 180), where *p* and *q* = 1 − *p* are the frequencies for the wild-type and deleterious allele, respectively, and *d* is the dominance effect which accounts for the deviation of the fitness value of the heterozygote from the average fitness of the two homozygotes (*d* = *s*(1 − 2*h*)/2, where *s* is the selection coefficient and *h* the dominance coefficient for each locus; [4], p. 44). The upper graph shows the decline in *B* across generations due to the loss of deleterious mutations by genetic drift and genetic purging selection. The red circle indicates the value of ΔID from *F*_*IBD*_ observed at generation 20. The lower graph shows the change in the average additive (*V*_*A*_) and dominance variance (*V*_*D*_) across generations for fitness, which were calculated as the sum over loci of 2*α*^2^*pq* and (2*dpq*)^2^, respectively, were *α* = *s*/2 + *d*(1 − 2*q*) is the average effect of an allelic substitution ([4], p. 44).

**Additional file 4: Table S2.** Statistical tests of the difference between mean *F* values from marker-based estimators and mean *F*_*IBD*_ values. Bootstraps are based on 1000 resamplings. ^1^Limits for 95% confidence intervals. ^2^Probability values < 0.001

**Additional file 5: Figure S3.** Inbreeding estimates, correlations with *F*_*IBD*_ and estimates of the rate of inbreeding depression for fitness obtained for a population of *N* = 20 breeding individuals maintained for 20 discrete generations assuming random mating and random contributions from parents to progeny (RC), equalization of contributions from parents to progeny (EC), and artificial selection for a neutral quantitative trait (SEL). Mean (*F*) and variance (*VF*) of inbreeding coefficients at generation 20, correlation between estimated inbreeding coefficients and those obtained from IBD measures (*r*), and mean values of the rate of inbreeding depression (ΔID). Bars refer to true IBD values (*F*_*IBD*_), and estimated from pedigree records (*F*_*PED*_) and from different marker-based measures (*F*_*VR*1_, *F*_*VR*2_, *F*_*YA*1_, *F*_*YA*2_, *F*_*LH*1_, *F*_*LH*2_, *F*_*HOM*_; see text for definitions) assuming the frequencies of the base generation (blue bars), those of the current generation (yellow bars) or a constant frequency of 0.5 (*F*_*q*05_; purple bars). Estimates from runs of homozygosity are shown for fragments longer than 1 Mb (*F*_*ROH*-1_) or 5 Mb (*F*_*ROH*-5_). Only subscripts of estimators are shown for the sake of clarity.

**Additional file 6: Figure S4.** Inbreeding estimates, correlations with *F*_*IBD*_ and estimates of the rate of inbreeding depression for fitness obtained for a population of *N* = 100 breeding individuals maintained for 50 discrete generations assuming random mating and random contributions from parents to progeny (RC), equalization of contributions from parents to progeny (EC), and artificial selection for a neutral quantitative trait (SEL). Mean (*F*) and variance (*VF*) of inbreeding coefficients at generation 50, correlation between estimated inbreeding coefficients and those obtained from IBD measures (*r*), and mean values of the rate of inbreeding depression (ΔID). Bars refer to true IBD values (*F*_*IBD*_), and estimated from pedigree records (*F*_*PED*_) and from different marker-based measures (*F*_*VR*1_, *F*_*VR*2_, *F*_*YA*1_, *F*_*YA*2_, *F*_*LH*1_, *F*_*LH*2_, *F*_*HOM*_; see text for definitions) assuming the frequencies of the base generation (blue bars), those of the current generation (yellow bars) or a constant frequency of 0.5 (*F*_*q*05_; purple bars). Estimates from runs of homozygosity are shown for fragments longer than 1 Mb (*F*_*ROH*-1_) or 5 Mb (*F*_*ROH*-5_). Only subscripts of estimators are shown for the sake of clarity.

**Additional file 7: Table S3.** Minimum and maximum values of the individual inbreeding coefficient (*F*) for populations of size *N* run for *t* generations assuming random mating and random contributions from parents to progeny (RC), equalization of contributions from parents to progeny (EC), and artificial selection for a neutral quantitative trait (SEL). Values refer to true IBD values (*F*_*IBD*_), and estimated from pedigree records (*F*_*PED*_) and from different marker-based measures (*F*_*VR*1_, *F*_*VR*2_, *F*_*YA*1_, *F*_*YA*2_, *F*_*LH*1_, *F*_*LH*2_, *F*_*HOM*_, *F*_*ROH*_; see text for definitions). The results correspond to those of Fig. 1, Additional file 5: Fig. S3 and Additional file 6: Fig. S4.

**Additional file 8: Figure S5.** Power to detect inbreeding depression obtained by counting the percentage of replicates where the value of ΔID was significantly different from zero with a 95% probability for a population of *N* breeding individuals maintained assuming random mating and random contributions from parents to progeny (RC), equalization of contributions from parents to progeny (EC), and artificial selection for a neutral quantitative trait (SEL). Populations with *N* = 20 run for 10 (**a**) or 20 (**b**) generations, and for *N* = 100 run for 50 generations (**c**). Bars refer to true IBD values (*F*_*IBD*_), and estimated from pedigree records (*F*_*PED*_) and from different marker-based measures (*F*_*VR*1_, *F*_*VR*2_, *F*_*YA*1_, *F*_*YA*2_, *F*_*LH*1_, *F*_*LH*2_, *F*_*HOM*_; see text for definitions) assuming the frequencies of the base generation (blue bars), those of the current generation (yellow bars) or a constant frequency of 0.5 (*F*_*q*05_; purple bars). Estimates from runs of homozygosity are shown for fragments longer than 1 Mb (*F*_*ROH*-1_) or 5 Mb (*F*_*ROH*-5_). Only subscripts of estimators are shown for the sake of clarity.

**Additional file 9: Figure S6.** Correlation between the values of inbreeding obtained with the different estimators when base population frequencies are known or those from the current generation are used assuming random mating and random contributions from parents to progeny (RC), equalization of contributions from parents to progeny (EC), and artificial selection for the quantitative trait (SEL) under a neutral model of variation for fitness. Populations of *N* = 20 breeding individuals maintained for 10 discrete generations (**a**), of *N* = 20 breeding individuals maintained for 20 discrete generations (**b**), and of *N* = 100 breeding individuals maintained for 50 discrete generations (**c**). In the first set of graphs (**a**–**c**) allele frequencies of the base population are assumed for the estimators. In the second set (**d**–**f**), current allele frequencies are assumed. Only subscripts of estimators are shown for the sake of clarity.

**Additional file 10: Figure S7. **Mean estimates of the inbreeding coefficient (*F*), variance of *F* values (*VF*), correlation (*r*) between *F* estimates and IBD measures, and estimates of the rate of inbreeding depression for fitness (ΔID) obtained for a population maintained with random mating and random contributions from parents to progeny (RC). **a** Scenario with *N* = 20 individuals run for 10 generations but considering a density of SNPs more than double of that in Fig. 1 (see Table 1). **b** Scenario with *N* = 20 individuals run for 10 generations assuming an alternative model of deleterious mutations where the mean effect of homozygous effects was 1/4 of that considered in the previous figures. **c** Scenario with *N* = 100 individuals run for 100 generations instead of 50. **d** Scenario with *N* = 20 individuals run for 20 generations where the base population is set up at generation 10. Bars refer to true IBD values (*F*_*IBD*_), and estimated from pedigree records (*F*_*PED*_) and from different marker-based measures (*F*_*VR*1_, *F*_*VR*2_, *F*_*YA*1_, *F*_*YA*2_, *F*_*LH*1_, *F*_*LH*2_, *F*_*HOM*_; see text for definitions) assuming the frequencies of the base generation (blue bars), those of the current generation (yellow bars) or a constant frequency of 0.5 (*F*_*q*05_; purple bars). Estimates from runs of homozygosity are shown for fragments longer than 1 Mb (*F*_*ROH*-1_) or 5 Mb (*F*_*ROH*-5_). Only subscripts of estimators are shown for the sake of clarity.

**Additional file 11: Figure S8.** Estimates of the rate of inbreeding depression (ΔID) for a sample of *N* = 100 individuals sampled from a large population.The estimates refer to different marker frequency-based measures (*F*_*VR*1_, *F*_*VR*2_, *F*_*YA*1_, *F*_*YA*2_, *F*_*LH*1_, *F*_*LH*2_; see text for definitions), assuming the frequencies of the current generation, estimates from homozygosity of SNPs (*F*_*HOM*_), and estimates from runs of homozygosity for fragments longer than 1 Mb (*F*_*ROH*-1_) or 5 Mb (*F*_*ROH*-5_). Estimates are obtained assuming minor allele frequencies (MAF) equal to 0 (red bars), 0.01 (orange bars) and 0.05 (green bars). The horizontal red line indicates the true ΔID in the population.

## Data Availability

All software and scripts used in the simulations are available in GitHub: https://github.com/armando-caballero/Molecular_Estimates_of_Inbreeding.

## Derivation of the estimator from Yang et al.

The estimator proposed by Yang et al. [20] is based on the correlation between the allele frequencies of uniting gametes. Let ${p}_{kg1}$ and ${p}_{kg2}$ be the allele frequencies for a biallelic locus $k$ of the two gametes producing a given individual $i$. Thus, ${p}_{kg1}$ and ${p}_{kg2}$ take values of 1 or 0 if the gametes carry or not a given allele of reference. The allele frequency of individual $i$ at the locus is thus:

1 $${p}_{ki}=\frac{{p}_{kg1}+{p}_{kg2}}{2},$$

which takes values 0, ½ or 1, if the individual carries no copies, one copy or two copies of the reference allele.

The correlation of allele frequencies across all $S$ loci for individual $i$ is:

2 $${r}_{UG}=\frac{1}{S}{\sum }_{k=1}^{S}\frac{({p}_{kg1}-{p}_{k})({p}_{kg2}-{p}_{k})}{{p}_{k}{q}_{k}},$$

where ${p}_{k}$ and ${q}_{k}=1-{p}_{k}$ are the average frequencies for the two alleles at locus $k$ in the whole population.

Substituting Eq. (1) into Eq. (2) we obtain:

$${r}_{UG}=\frac{1}{S}{\sum }_{k=1}^{S}\frac{(2{p}_{ki}-{p}_{kg2}-{p}_{k})(2{p}_{ki}-{p}_{kg1}-{p}_{k})}{{p}_{k}{q}_{k}}=\frac{1}{S}{\sum }_{k=1}^{S}\frac{{(2{p}_{ki}-{p}_{k})}^{2}-\left(2{p}_{ki}-{p}_{k}\right){p}_{kg1}-\left(2{p}_{ki}-{p}_{k}\right){p}_{kg2}+{p}_{kg1}{p}_{kg2}}{{p}_{k}{q}_{k}}=\frac{1}{S}{\sum }_{k=1}^{S}\frac{\left(2{p}_{ki}-{p}_{k}\right)\left[\left(2{p}_{ki}-{p}_{k}\right)-\left({p}_{kg1}+{p}_{kg2}\right)\right]+{p}_{kg1}{p}_{kg2}}{{p}_{k}{q}_{k}}=\frac{1}{S}{\sum }_{k=1}^{S}\frac{-\left(2{p}_{ki}-{p}_{k}\right){p}_{k}+{p}_{kg1}{p}_{kg2}}{{p}_{k}{q}_{k}}.$$

Adding and subtracting $2{p}_{ki}^{2}$ in the numerator and operating, ${r}_{UG}=\frac{1}{S}{\sum }_{k=1}^{S}\frac{{({p}_{ki}-{p}_{k})}^{2}+{p}_{ki}^{2}-2{\left(\frac{{p}_{kg1}+{p}_{kg2}}{2}\right)}^{2}+{p}_{kg1}{p}_{kg2}}{{p}_{k}{q}_{k}}=\frac{1}{S}{\sum }_{k=1}^{S}\frac{{({p}_{ki}-{p}_{k})}^{2}+{p}_{ki}^{2}-\left(\frac{{p}_{kg1}^{2}+{p}_{kg2}^{2}}{2}\right)}{{p}_{k}{q}_{k}}$

Because, ${p}_{kg1}^{2}={p}_{kg1}$ and ${p}_{kg2}^{2}={p}_{kg2}$, then,

3 $${r}_{UG}=\frac{1}{S}{\sum }_{k=1}^{S}\frac{{({p}_{ki}-{p}_{k})}^{2}-{p}_{ki}(1-{p}_{ki})}{{p}_{k}{q}_{k}}.$$

Equation (3), which was also shown in the Appendix of Caballero et al. [32], indicates that the estimator is based on the squared deviation of the allele frequencies of individuals from the population mean minus the expected variance within individuals. This contrasts with the estimator of VanRaden [19], which is based on the correlation of allele frequencies between individuals and does not include the correction for the expected variance within individuals in the numerator of its expression,

4 $${F}_{VR1}=\frac{{\sum }_{k=1}^{S}2{({p}_{ki}-{p}_{k})}^{2}}{{\sum }_{k=1}^{S}{p}_{k}{q}_{k}}-1.$$

(see Appendix of Caballero et al. [32]).

After some algebra on Eq. (3),

$${r}_{UG}=\frac{1}{S}{\sum }_{k=1}^{S}\frac{4{p}_{ki}^{2}-2{p}_{ki}\left(1+2{p}_{k}\right)+2{p}_{k}^{2}}{{p}_{k}{q}_{k}}.$$

And taking ${x}_{k}=2{p}_{ki}$ as the number of alleles of reference, i.e. ${x}_{k}$ takes values 0, 1 or 2 for individual $i$,

5 $${F}_{YA2}={r}_{UG}=\frac{1}{S}\sum_{k=1}^{S}\frac{{x}_{k}^{2}-\left(1+2{p}_{k}\right){x}_{k}+2{p}_{k}^{2}}{2{p}_{k}{q}_{k}},$$

which is the equation shown in the main text for this estimator. Using the same change of terms, Eq. (4) becomes the equation for ${F}_{VR1}$ of the main text.
