# Supplementary figures and images for: A comparison of marker-based estimators of inbreeding and inbreeding depression
Source: Genet Sel Evol. 2022 Dec 27;54:82. doi: 10.1186/s12711-022-00772-0 (PMC9793638; doi:10.1186/s12711-022-00772-0)

**Fig. S1**

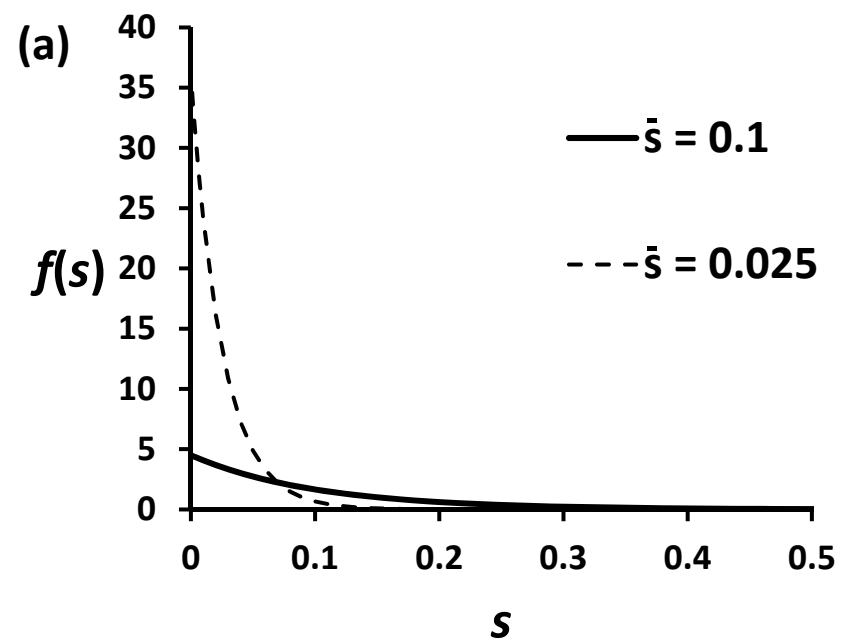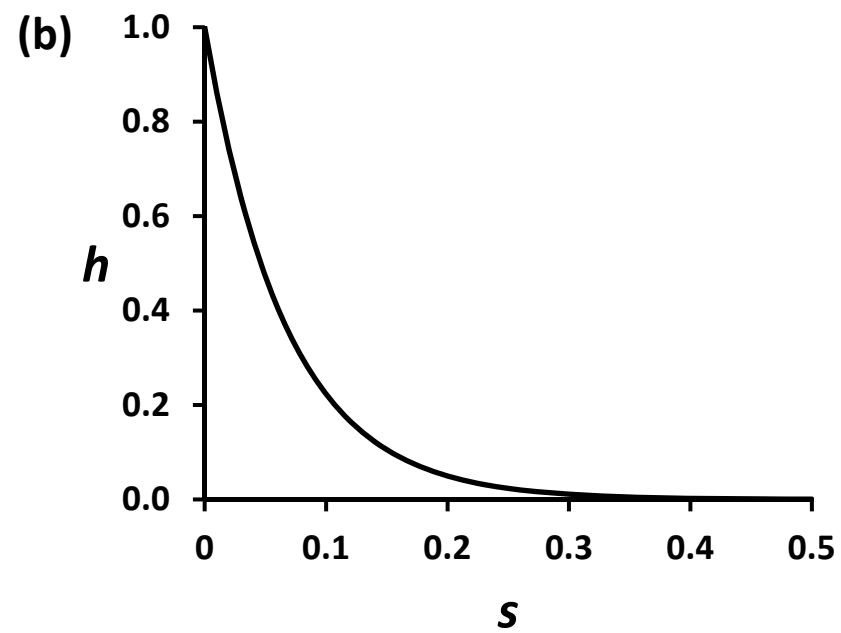

Supplement: Supplementary file 1 — Additional file 1: Figure S1. Distribution of mutational homozygous effects and dominance coefficients assumed in the simulations. a Homozygous effects (s) had an exponential distribution with mean effect $\overline{s }$s¯ = 0.1 or 0.025. The number of mutations are scaled by the haploid mutation rate in each model. b Dominance coefficients (h) were assumed to have an inverse relationship with s values and were taken from a uniform distribution between 0 and e(−ks), where k is a constant needed to get an average value of $\overline{h }$h¯ = 0.2. [file 12711_2022_772_MOESM1_ESM.pdf]

Fig. S2

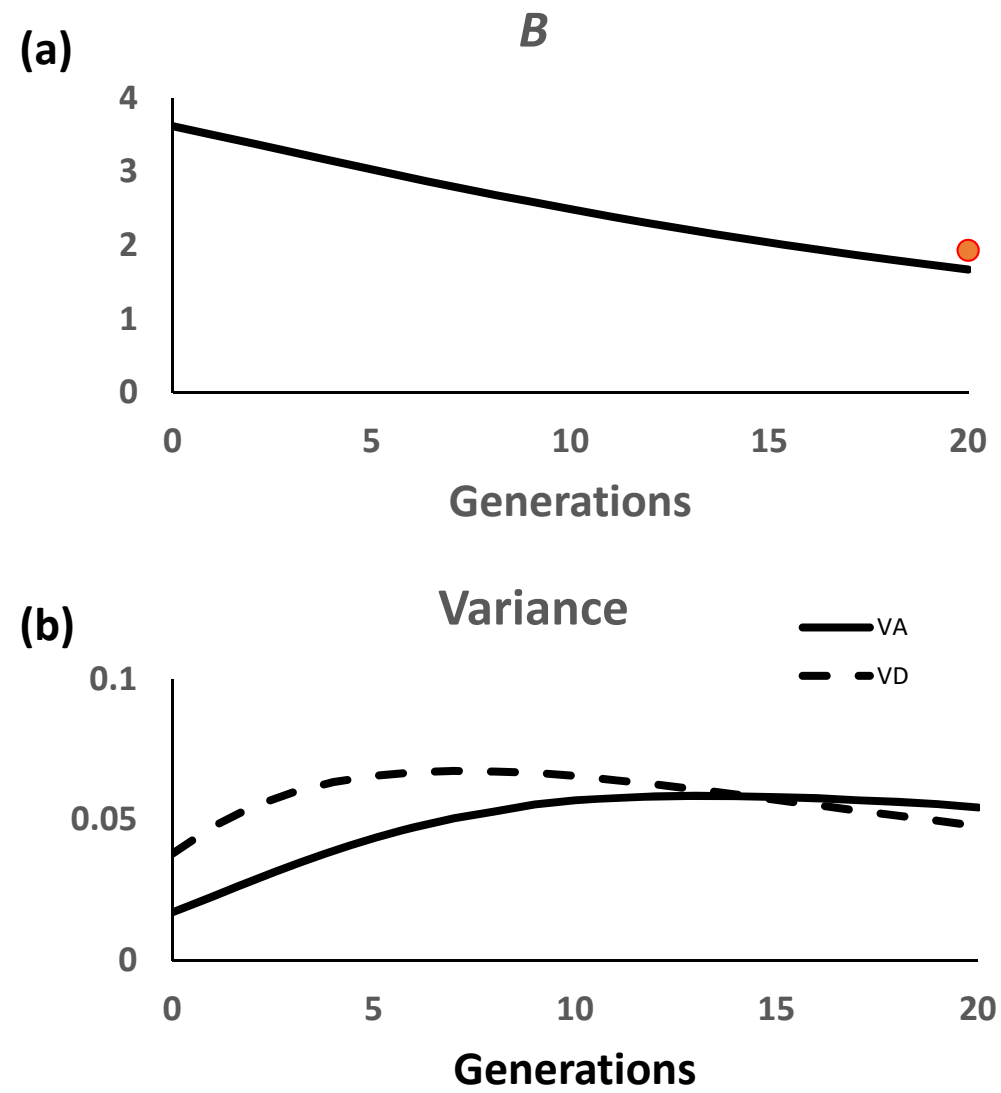

Supplement: Supplementary file 3 — Additional file 3: Figure S2. Average inbreeding load (B), additive (VA) and dominance (VD) variances for fitness for a population of N = 20 breeding individuals maintained for 20 discrete generations assuming random mating and random contributions from parents to progeny (RC). The inbreeding load (B) measures the cumulative deleterious effect of (partially) recessive mutations that is hidden in large non-inbred populations and is expressed by inbreeding and is quantified in terms of number of lethal equivalents per haploid genome. In the absence of selection, B equals the rate of inbreeding depression (ΔID). The values of B in the simulations were calculated as the sum over loci of 2dpq ([4], p. 180), where p and q = 1 − p are the frequencies for the wild-type and deleterious allele, respectively, and d is the dominance effect which accounts for the deviation of the fitness value of the heterozygote from the average fitness of the two homozygotes (d = s(1 − 2h)/2, where s is the selection coefficient and h the dominance coefficient for each locus; [4], p. 44). The upper graph shows the decline in B across generations due to the loss of deleterious mutations by genetic drift and genetic purging selection. The red circle indicates the value of ΔID from FIBD observed at generation 20. The lower graph shows the change in the average additive (VA) and dominance variance (VD) across generations for fitness, which were calculated as the sum over loci of 2α2pq and (2dpq)2, respectively, were α = s/2 + d(1 − 2q) is the average effect of an allelic substitution ([4], p. 44). [file 12711_2022_772_MOESM3_ESM.pdf]

Fig. S3

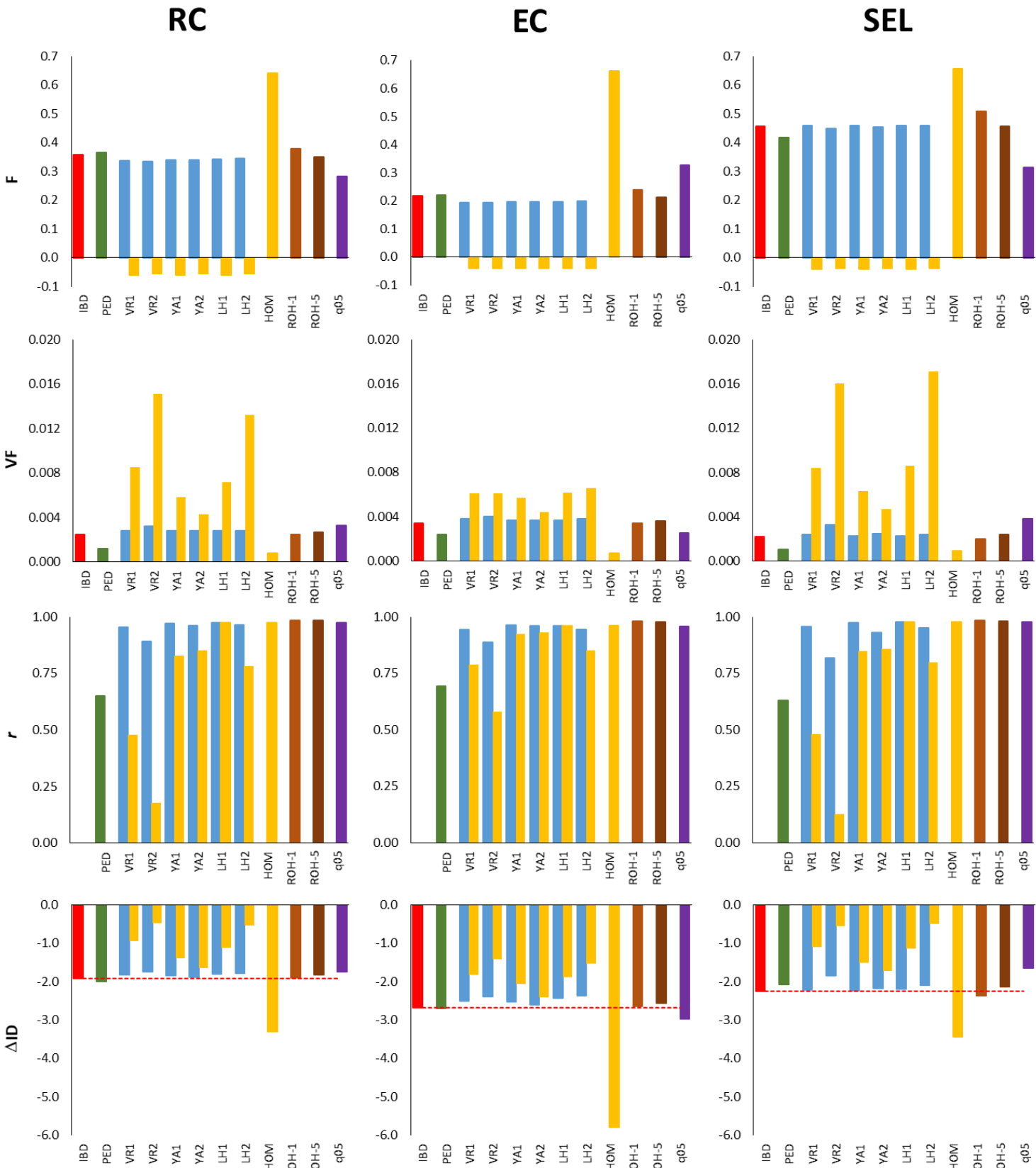

Supplement: Supplementary file 5 — Additional file 5: Figure S3. Inbreeding estimates, correlations with FIBD and estimates of the rate of inbreeding depression for fitness obtained for a population of N = 20 breeding individuals maintained for 20 discrete generations assuming random mating and random contributions from parents to progeny (RC), equalization of contributions from parents to progeny (EC), and artificial selection for a neutral quantitative trait (SEL). Mean (F) and variance (VF) of inbreeding coefficients at generation 20, correlation between estimated inbreeding coefficients and those obtained from IBD measures (r), and mean values of the rate of inbreeding depression (ΔID). Bars refer to true IBD values (FIBD), and estimated from pedigree records (FPED) and from different marker-based measures (FVR1, FVR2, FYA1, FYA2, FLH1, FLH2, FHOM; see text for definitions) assuming the frequencies of the base generation (blue bars), those of the current generation (yellow bars) or a constant frequency of 0.5 (Fq05; purple bars). Estimates from runs of homozygosity are shown for fragments longer than 1 Mb (FROH-1) or 5 Mb (FROH-5). Only subscripts of estimators are shown for the sake of clarity. [file 12711_2022_772_MOESM5_ESM.pdf]

Fig. S4

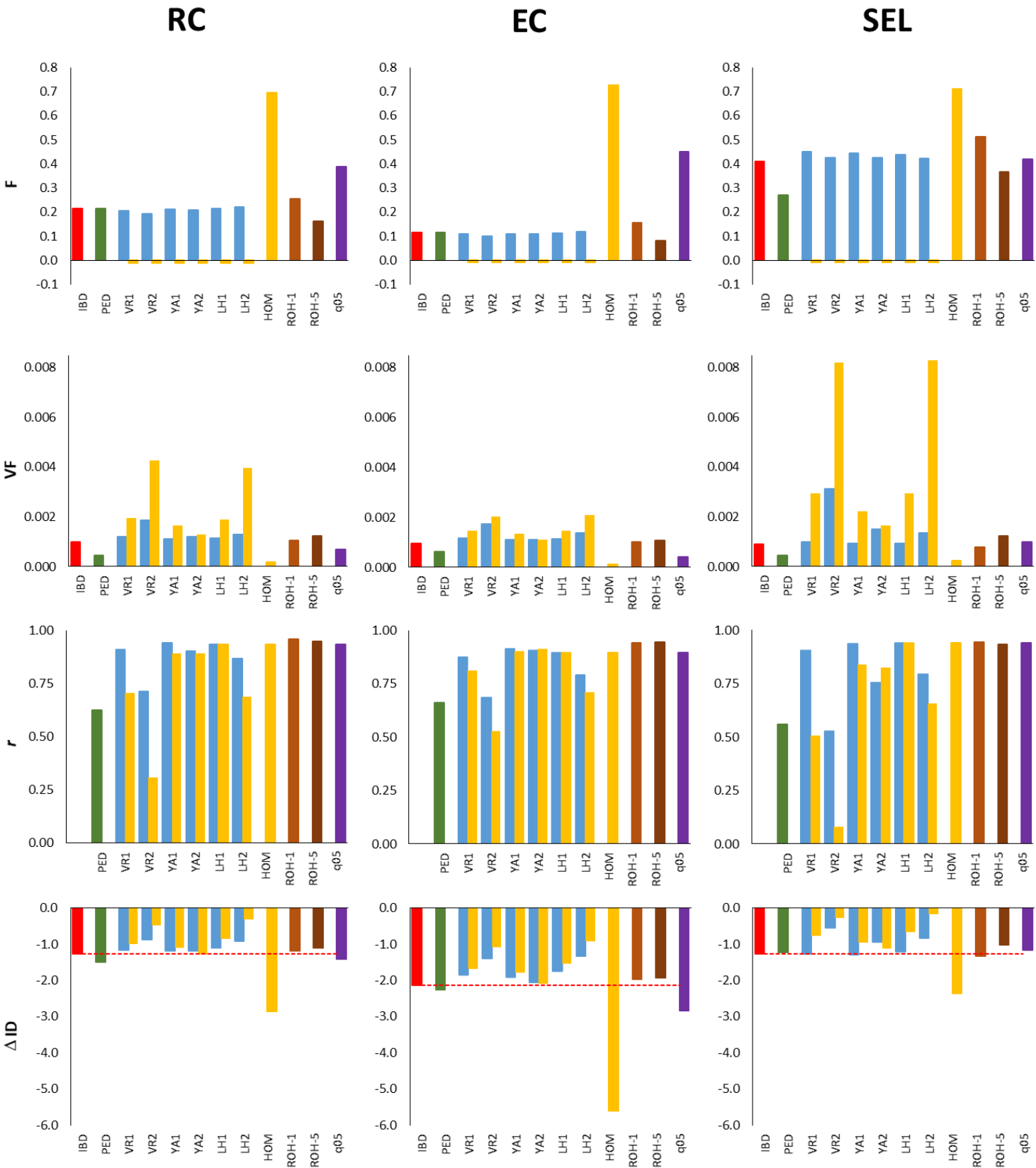

Supplement: Supplementary file 6 — Additional file 6: Figure S4. Inbreeding estimates, correlations with FIBD and estimates of the rate of inbreeding depression for fitness obtained for a population of N = 100 breeding individuals maintained for 50 discrete generations assuming random mating and random contributions from parents to progeny (RC), equalization of contributions from parents to progeny (EC), and artificial selection for a neutral quantitative trait (SEL). Mean (F) and variance (VF) of inbreeding coefficients at generation 50, correlation between estimated inbreeding coefficients and those obtained from IBD measures (r), and mean values of the rate of inbreeding depression (ΔID). Bars refer to true IBD values (FIBD), and estimated from pedigree records (FPED) and from different marker-based measures (FVR1, FVR2, FYA1, FYA2, FLH1, FLH2, FHOM; see text for definitions) assuming the frequencies of the base generation (blue bars), those of the current generation (yellow bars) or a constant frequency of 0.5 (Fq05; purple bars). Estimates from runs of homozygosity are shown for fragments longer than 1 Mb (FROH-1) or 5 Mb (FROH-5). Only subscripts of estimators are shown for the sake of clarity. [file 12711_2022_772_MOESM6_ESM.pdf]

Fig. S5

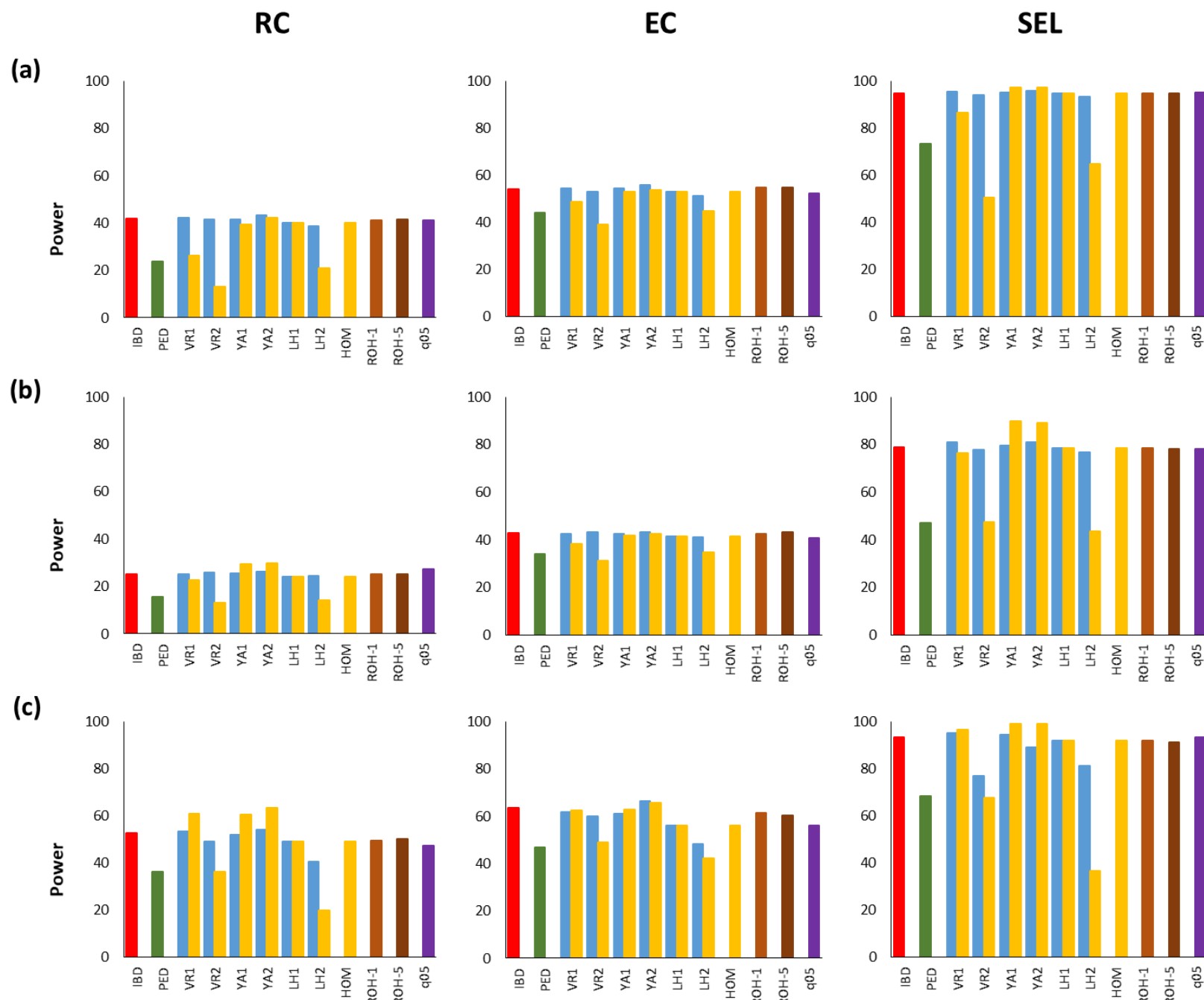

Supplement: Supplementary file 8 — Additional file 8: Figure S5. Power to detect inbreeding depression obtained by counting the percentage of replicates where the value of ΔID was significantly different from zero with a 95% probability for a population of N breeding individuals maintained assuming random mating and random contributions from parents to progeny (RC), equalization of contributions from parents to progeny (EC), and artificial selection for a neutral quantitative trait (SEL). Populations with N = 20 run for 10 (a) or 20 (b) generations, and for N = 100 run for 50 generations (c). Bars refer to true IBD values (FIBD), and estimated from pedigree records (FPED) and from different marker-based measures (FVR1, FVR2, FYA1, FYA2, FLH1, FLH2, FHOM; see text for definitions) assuming the frequencies of the base generation (blue bars), those of the current generation (yellow bars) or a constant frequency of 0.5 (Fq05; purple bars). Estimates from runs of homozygosity are shown for fragments longer than 1 Mb (FROH-1) or 5 Mb (FROH-5). Only subscripts of estimators are shown for the sake of clarity. [file 12711_2022_772_MOESM8_ESM.pdf]

Fig. S6

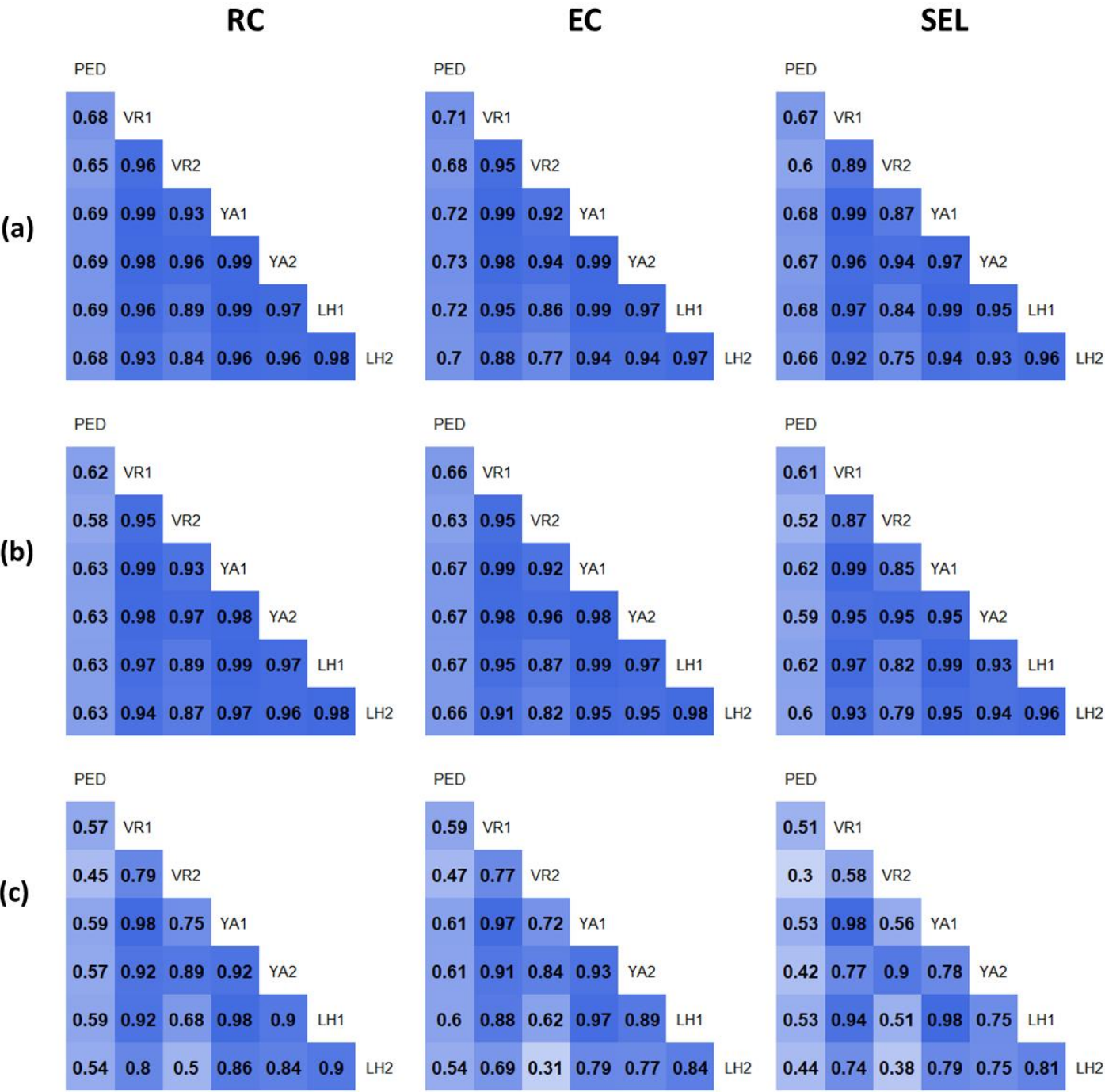

RC

EC

SEL

(d)

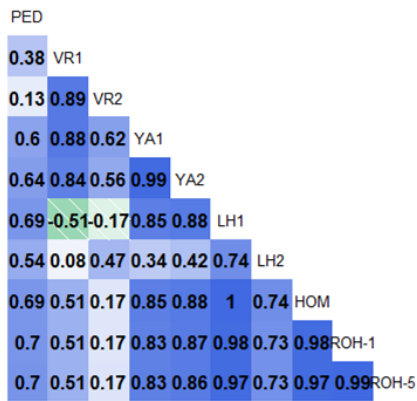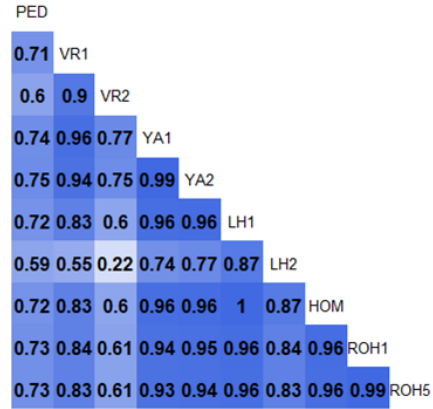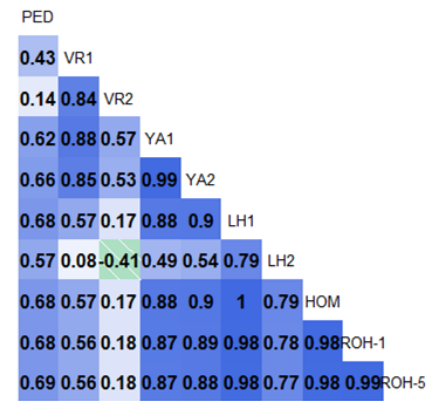

(e)

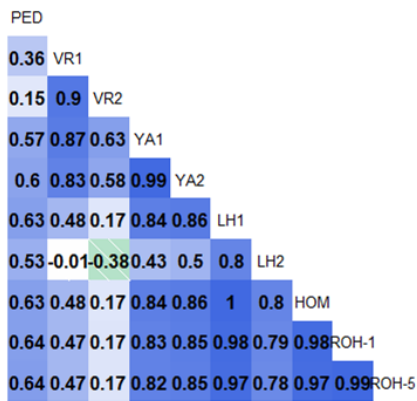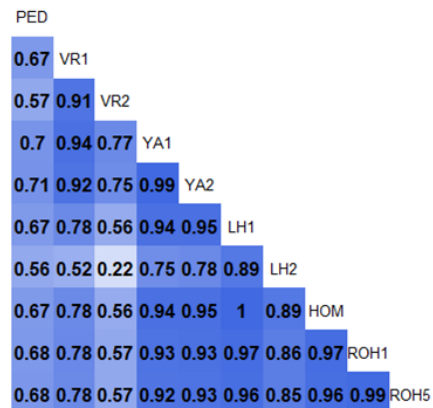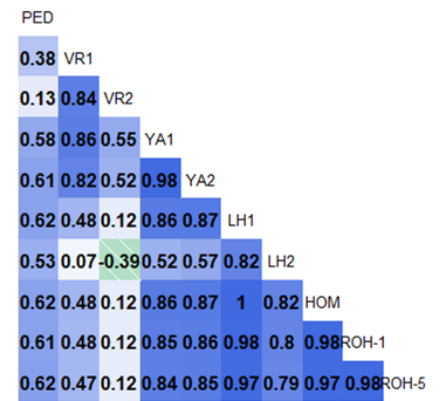

(f)

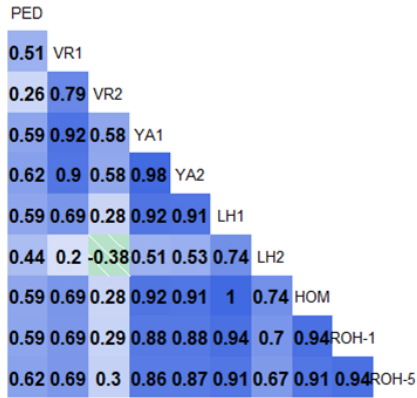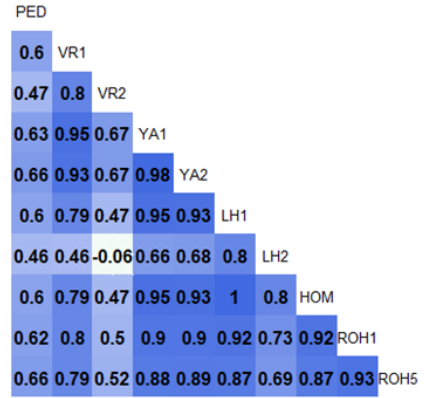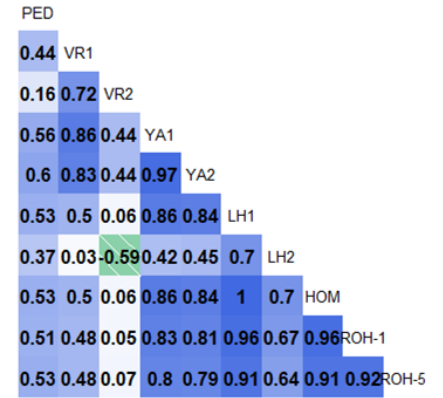

Supplement: Supplementary file 9 — Additional file 9: Figure S6. Correlation between the values of inbreeding obtained with the different estimators when base population frequencies are known or those from the current generation are used assuming random mating and random contributions from parents to progeny (RC), equalization of contributions from parents to progeny (EC), and artificial selection for the quantitative trait (SEL) under a neutral model of variation for fitness. Populations of N = 20 breeding individuals maintained for 10 discrete generations (a), of N = 20 breeding individuals maintained for 20 discrete generations (b), and of N = 100 breeding individuals maintained for 50 discrete generations (c). In the first set of graphs (a–c) allele frequencies of the base population are assumed for the estimators. In the second set (d–f), current allele frequencies are assumed. Only subscripts of estimators are shown for the sake of clarity. [file 12711_2022_772_MOESM9_ESM.pdf]

**Fig. S7**

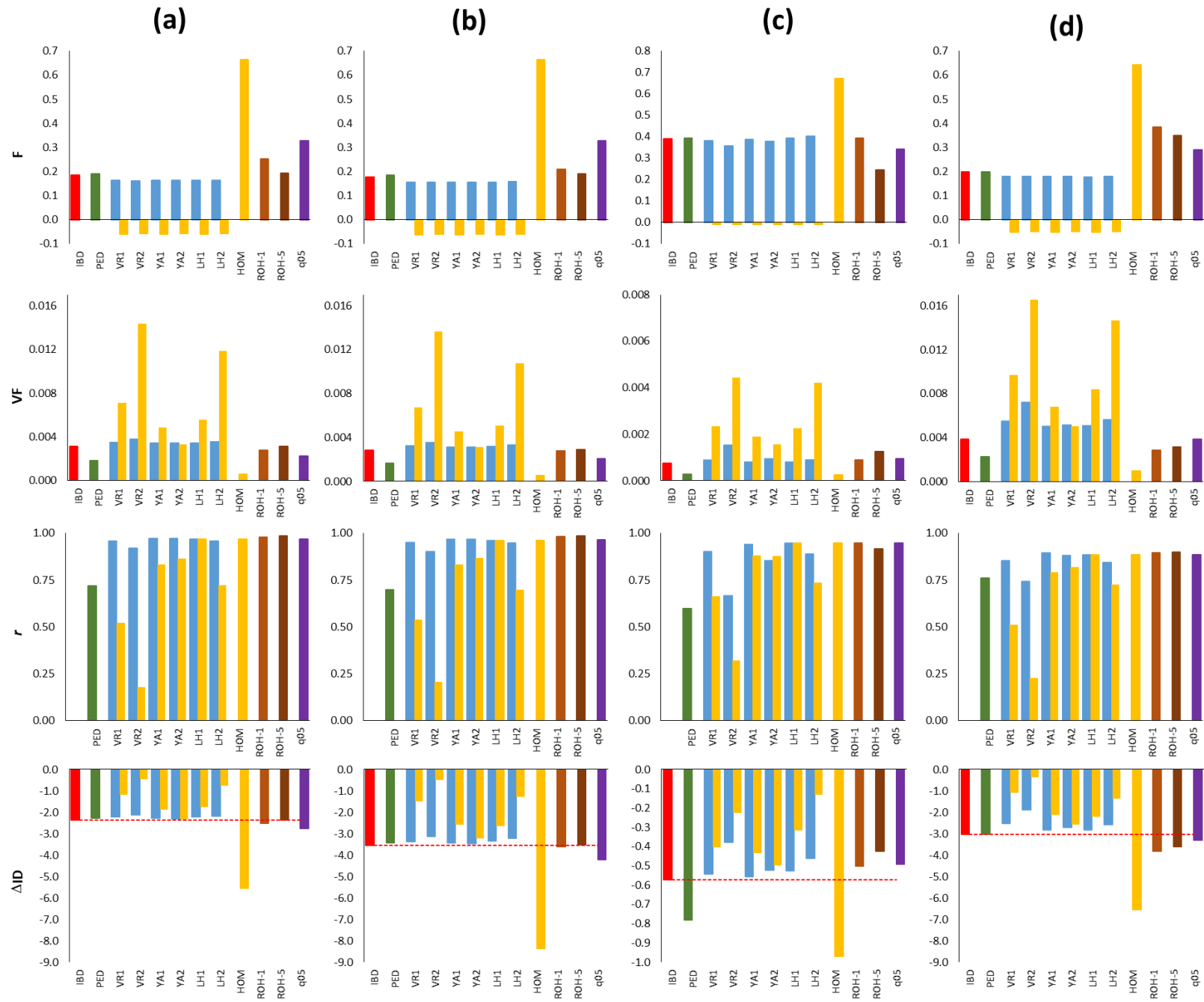

Supplement: Supplementary file 10 — Additional file 10: Figure S7. Mean estimates of the inbreeding coefficient (F), variance of F values (VF), correlation (r) between F estimates and IBD measures, and estimates of the rate of inbreeding depression for fitness (ΔID) obtained for a population maintained with random mating and random contributions from parents to progeny (RC). a Scenario with N = 20 individuals run for 10 generations but considering a density of SNPs more than double of that in Fig. 1 (see Table 1). b Scenario with N = 20 individuals run for 10 generations assuming an alternative model of deleterious mutations where the mean effect of homozygous effects was 1/4 of that considered in the previous figures. c Scenario with N = 100 individuals run for 100 generations instead of 50. d Scenario with N = 20 individuals run for 20 generations where the base population is set up at generation 10. Bars refer to true IBD values (FIBD), and estimated from pedigree records (FPED) and from different marker-based measures (FVR1, FVR2, FYA1, FYA2, FLH1, FLH2, FHOM; see text for definitions) assuming the frequencies of the base generation (blue bars), those of the current generation (yellow bars) or a constant frequency of 0.5 (Fq05; purple bars). Estimates from runs of homozygosity are shown for fragments longer than 1 Mb (FROH-1) or 5 Mb (FROH-5). Only subscripts of estimators are shown for the sake of clarity. [file 12711_2022_772_MOESM10_ESM.pdf]

Fig. S8

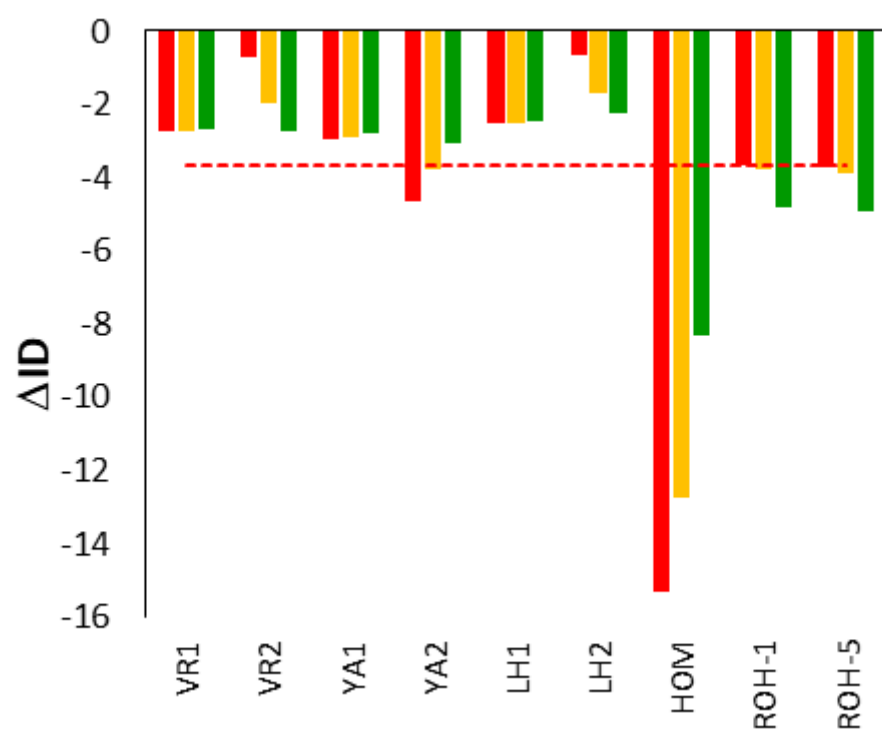

Supplement: Supplementary file 11 — Additional file 11: Figure S8. Estimates of the rate of inbreeding depression (ΔID) for a sample of N = 100 individuals sampled from a large population.The estimates refer to different marker frequency-based measures (FVR1, FVR2, FYA1, FYA2, FLH1, FLH2; see text for definitions), assuming the frequencies of the current generation, estimates from homozygosity of SNPs (FHOM), and estimates from runs of homozygosity for fragments longer than 1 Mb (FROH-1) or 5 Mb (FROH-5). Estimates are obtained assuming minor allele frequencies (MAF) equal to 0 (red bars), 0.01 (orange bars) and 0.05 (green bars). The horizontal red line indicates the true ΔID in the population. [file 12711_2022_772_MOESM11_ESM.pdf]
