# Supplementary material for: A comparison of marker-based estimators of inbreeding and inbreeding depression
Source: Genet Sel Evol. 2022 Dec 27;54:82. doi: 10.1186/s12711-022-00772-0 (PMC9793638; doi:10.1186/s12711-022-00772-0)
Supplement: Supplementary file 2 — Additional file 2: Table S1. Number of deleterious loci at generation 0 and those lost, fixed or segregating at generation 20 and their average frequency, for a range of selection coefficients (s). The results refer to a simulation with N = 20 individuals carried for 20 generations with scheme RC. [file 12711_2022_772_MOESM2_ESM.pdf]

**Table S1. Number of deleterious loci at generations 0 and those lost, fixed or segregating at generation 20 and their average frequency, for a range of selection coefficients (*s*)**

| **Range of *s* values** | **Generation 0** | | **Generation 20** | | |
|---|---|---|---|---|---|
| | **No.** | **Freq.** | **No. Lost (Fixed)** | **No. segregating** | **Freq.** |
| **0.0001 – 0.01** | 250 | 0.142 | 162 (5) | 83 | 0.364 |
| **0.0001 – 0.05** | 352 | 0.059 | 282 (0) | 70 | 0.589 |
| **0.05 – 0.15** | 355 | 0.043 | 286 (0) | 69 | 0.271 |
| **0.15 – 0.3** | 195 | 0.043 | 171 (0) | 24 | 0.270 |
| **0.3 – 1** | 47 | 0.041 | 47 (0) | 0 | - |

The results refer to a simulation with $N = 20$ individuals carried for 20 generations with scheme RC.
