# Supplementary material for: A comparison of marker-based estimators of inbreeding and inbreeding depression
Source: Genet Sel Evol. 2022 Dec 27;54:82. doi: 10.1186/s12711-022-00772-0 (PMC9793638; doi:10.1186/s12711-022-00772-0)
Supplement: Supplementary file 4 — Additional file 4: Table S2. Statistical tests of the difference between mean F values from marker-based estimators and mean FIBD values. Bootstraps are based on 1000 resamplings. 1Limits for 95% confidence intervals. 2Probability values < 0.001 [file 12711_2022_772_MOESM4_ESM.pdf]

Table S2. Statistical tests of the difference between mean *F* values from marker-based estimators and mean F_{IBD} values

	OBSERVED DIFFERENCE	Bootstrap limits[1] lower limit	upper limit	Probabilities[2] Bootstrap	t-test
**RC, frequencies from *t* = 0**					
PED	-0.1587	-0.2688	-0.0589	0	0
VR1	-0.1557	-0.1863	-0.1245	0	0
VR2	-0.2593	-0.3109	-0.2124	0	0
YA1	-0.1394	-0.1671	-0.1134	0	0
YA2	-0.0837	-0.1119	-0.0572	0	0
LH1	-0.2021	-0.2314	-0.1750	0	0
LH2	-0.2748	-0.3124	-0.2382	0	0
HOM	3.2496	3.1152	3.3964	0	0
ROH-1	0.0049	-0.0148	0.0269	0.3	0.506
ROH-5	-0.0356	-0.0547	-0.0155	0	0
q05	0.4111	0.3737	0.4499	0	0
**EC, frequencies from *t* = 0**					
PED	-0.0831	-0.2569	0.0845	0.183	0.18
VR1	-0.2701	-0.3187	-0.2229	0	0
VR2	-0.4400	-0.5058	-0.3762	0	0
YA1	-0.2170	-0.2615	-0.1721	0	0
YA2	-0.1085	-0.1442	-0.0703	0	0
LH1	-0.2991	-0.3528	-0.2499	0	0
LH2	-0.4202	-0.4805	-0.3536	0	0
HOM	5.1937	4.9609	5.4195	0	0
ROH-1	-0.0085	-0.0446	0.0291	0.314	0.528
ROH-5	-0.0442	-0.0785	-0.0107	0.003	0
q05	0.3030	0.2872	0.3184	0	0
**SEL, frequencies from *t* = 0**					
PED	-0.1848	-0.2286	-0.1399	0	0
VR1	-0.0829	-0.0972	-0.0674	0	0
VR2	-0.4697	-0.4982	-0.4381	0	0
YA1	-0.0627	-0.0745	-0.0500	0	0
YA2	-0.0602	-0.0760	-0.0422	0	0
LH1	-0.1117	-0.1241	-0.0989	0	0
LH2	-0.2735	-0.2916	-0.2565	0	0
HOM	3.2474	3.1812	3.3158	0	0

ROH-1	0.1136	0.1042	0.1238	0	0
ROH-5	-0.0292	-0.0369	-0.0215	0	0
q05	0.3030	0.2872	0.3184	0	0

		**Bootstrap limits[1]**			
	**OBSERVED**	**lower**	**upper**	**Probabilities[2]**	
	**DIFFERENCE**	**limit**	**limit**	**Bootstrap**	**t-test**
**RC, frequencies from *t* = 10**					
VR1	-1.3112	-1.4079	-1.2222	0	0
VR2	-2.0549	-2.1659	-1.9530	0	0
YA1	-0.5872	-0.6544	-0.5274	0	0
YA2	-0.1097	-0.1746	-0.0509	0	0
LH1	-0.6929	-0.7293	-0.6584	0	0
LH2	-1.7094	-1.7832	-1.6373	0	0
**EC, frequencies from *t* = 10**					
VR1	-0.8000	-0.8733	-0.7277	0	0
VR2	-1.3265	-1.4357	-1.2213	0	0
YA1	-0.5219	-0.5743	-0.4711	0	0
YA2	0.0270	-0.0247	0.0802	0.163	0.15
LH1	-0.6423	-0.6974	-0.5909	0	0
LH2	-1.0638	-1.1550	-0.9737	0	0
**SEL, frequencies from *t* = 10**					
VR1	-1.0502	-1.0979	-0.9995	0	0
VR2	-1.9432	-1.9988	-1.8868	0	0
YA1	-0.4388	-0.4690	-0.4104	0	0
YA2	-0.0421	-0.0693	-0.0138	0.003	0
LH1	-0.7689	-0.7861	-0.7495	0	0
LH2	-1.7936	-1.8295	-1.7574	0	0

Bootstraps are based on 1,000 resamplings. [1] Limits for 95% confidence intervals.

[2] A value of 0 indicates probability < 0.001
