# Supplementary material for: A comparison of marker-based estimators of inbreeding and inbreeding depression
Source: Genet Sel Evol. 2022 Dec 27;54:82. doi: 10.1186/s12711-022-00772-0 (PMC9793638; doi:10.1186/s12711-022-00772-0)
Supplement: Supplementary file 7 — Additional file 7: Table S3. Minimum and maximum values of the individual inbreeding coefficient (F) for populations of size N run for t generations assuming random mating and random contributions from parents to progeny (RC), equalization of contributions from parents to progeny (EC), and artificial selection for a neutral quantitative trait (SEL). Values refer to true IBD values (FIBD), and estimated from pedigree records (FPED) and from different marker-based measures (FVR1, FVR2, FYA1, FYA2, FLH1, FLH2, FHOM, FROH; see text for definitions). The results correspond to those of Fig. 1, Additional file 5: Fig. S3 and Additional file 6: Fig. S4. [file 12711_2022_772_MOESM7_ESM.pdf]

Table S3. Minimum and maximum values of the individual inbreeding coefficient (*F*) for populations of size *N* run for *t* generations assuming random mating and random contributions from parents to progeny (RC), equalization of contributions from parents to progeny (EC), and artificial selection for a neutral quantitative trait (SEL).

$N = 20, t = 10$		F_{IBD}	F_{PED}	F_{VR1}	F_{VR2}	F_{YA1}	F_{YA2}	F_{LH1}	F_{LH2}	F_{HOM}	$F_{ROH\text{-}1}$	$F_{ROH\text{-}5}$
RC												
	Min	0.039	0.097	-0.345	-0.423	-0.264	-0.203	-0.267	-0.579	0.590	0.070	0.054
	Max	0.551	0.473	0.424	0.745	0.337	0.321	0.395	0.479	0.806	0.581	0.561
EC												
	Min	0.007	0.046	-0.207	-0.229	-0.183	-0.144	-0.190	-0.253	0.643	0.031	0.012
	Max	0.636	0.547	0.554	0.534	0.551	0.483	0.560	0.512	0.869	0.655	0.644
SEL												
	Min	0.052	0.115	-0.343	-0.377	-0.273	-0.216	-0.305	-0.848	0.581	0.116	0.067
	Max	0.628	0.514	0.498	0.914	0.432	0.400	0.492	0.509	0.841	0.667	0.649
$N = 20, t = 20$												
RC												
	Min	0.188	0.270	-0.382	-0.427	-0.279	-0.235	-0.314	-0.687	0.547	0.191	0.163
	Max	0.657	0.631	0.431	0.582	0.373	0.375	0.416	0.489	0.804	0.678	0.662
EC												
	Min	0.068	0.156	-0.218	-0.253	-0.215	-0.170	-0.223	-0.320	0.601	0.099	0.071
	Max	0.623	0.579	0.521	0.601	0.491	0.457	0.502	0.507	0.840	0.641	0.627
SEL												
	Min	0.244	0.310	-0.349	-0.412	-0.296	-0.257	-0.378	-1.529	0.542	0.311	0.246
	Max	0.737	0.635	0.601	1.366	0.514	0.500	0.491	0.552	0.834	0.764	0.744
$N = 100, t = 50$												
RC												
	Min	0.118	0.187	-0.151	-0.225	-0.127	-0.108	-0.149	-0.372	0.652	0.146	0.060
	Max	0.576	0.460	0.484	0.464	0.481	0.458	0.477	0.453	0.841	0.613	0.575
EC												
	Min	0.052	0.102	-0.114	-0.142	-0.104	-0.084	-0.115	-0.188	0.696	0.070	0.015
	Max	0.542	0.447	0.456	0.525	0.465	0.468	0.474	0.456	0.856	0.580	0.522
SEL												
	Min	0.275	0.231	-0.217	-0.300	-0.178	-0.133	-0.234	-1.557	0.644	0.381	0.198
	Max	0.697	0.499	0.449	1.685	0.417	0.469	0.460	0.481	0.844	0.747	0.679

Values refer to true IBD values (F_{IBD}), and estimated from pedigree records (F_{PED}) and from different marker-based measures (F_{VR1}, F_{VR2}, F_{YA1}, F_{YA2}, F_{LH1}, F_{LH2}, F_{HOM}, F_{ROH}; see text for definitions). The results correspond to those of Figures 1, S3 and S4.
